# Lipoxygenase Inhibitory Activity and Prostate Cancer Cytotoxicity of In Situ- and In Vitro-Cultivated Balkan Endemic *Sideritis scardica* Griseb

**DOI:** 10.3390/plants14213263

**Published:** 2025-10-25

**Authors:** Kalina Danova, Jasmina Petreska Stanoeva, Elena Stoyanova, Kalina Alipieva, Marina Stefova, Ina Aneva

**Affiliations:** 1 Institute of Organic Chemistry with Centre of Phytochemistry, Bulgarian Academy of Sciences, Acad. Georgi Bonchev Str., bl.9, 1113 Sofia, Bulgaria; kalina.alipieva@orgchm.bas.bg; 2 Institute of Chemistry, Faculty of Natural Science and Mathematics, Ss. Cyril and Methodius University in Skopje, Arhimedova 5, 1000 Skopje, North Macedonia; jasmina.petreska@pmf.ukim.mk (J.P.S.); marinaiv@pmf.ukim.mk (M.S.); 3 Institute of Biology and Immunology of Reproduction “Acad. Kiril Bratanov”, Bulgarian Academy of Sciences, 73 Tzarigradsko Shose Blvd., 1113 Sofia, Bulgaria; elena.n.st@gmail.com; 4 Institute of Biodiversity and Ecosystem Research, Bulgarian Academy of Sciences, 2 Gagarin Str., 1113 Sofia, Bulgaria; ina.aneva@abv.bg

**Keywords:** *Sideritis scardica* Griseb, wild accessions, in vitro culture, 15-lypoxigenase inhibition, prostate cancer cytotoxicity, WPMW-1 cell line, PC-3 cell line, LNCaP cell line, phenylethanoids, flavone glycosides

## Abstract

*Sideritis scardica* Griseb., a Balkan endemic species traditionally used for respiratory conditions and wound-healing, was investigated for its 15-lipoxygenase (15-LOX) inhibitory and cytotoxic activities in relation to its phenolic composition. Aerial parts from the wild-collected and in vitro-cultivated plant were successively extracted with hexane, chloroform, and methanol and the methanol extract was further fractionated into ethyl acetate, butanol, and water fractions. This study presents the first combined evaluation of LOX inhibition and cytotoxicity against prostate cell lines WPMY-1 (normal epithelial fibroblast/myofibroblast), PC-3 (epithelial adenocarcinoma, Grade IV), and LNCaP (epithelial carcinoma) and detailed phytochemical profiles of wild-collected and in vitro-cultivated *S. scardica*. The phytochemical analysis revealed distinct profiles: increased LOX-inhibition activity was related to a higher phenylethanoid/flavone glycoside ratio, while PC cytotoxicity was rather related to flavone glycoside domination in the plant preparations. The cytotoxic effect of the in vitro-obtained preparations was characterized by a marked selectivity when comparing their effects on WPMY-1, PC-3 and LNCaP cells. To our knowledge, this is the first report linking phenylethanoid/flavone glycoside profiles of in situ and in vitro *S. scardica* plants with dual LOX-inhibitory and anti-prostate cancer activities, supporting the plant’s potential as a sustainable source of bioactive compounds.

## 1. Introduction

Oncological ailments are an expression of a complex and multifunctional disorder of physiological processes in the human organism. Manifested through the uncontrolled growth of cells, which can invade and spread to distant body locations, they are among the leading causes of illness and mortality worldwide, with approximately 9.6 million deaths in 2018. Lung, prostate, colorectal, stomach, and liver cancer are the most common types of cancer in men, while breast, colorectal, lung, cervical, and thyroid cancer are the most common among women [1]. Treatment options include surgery, chemotherapy, and radiotherapy, tailored to tumor stage, type, and available resources.

The direct cytotoxicity of certain plant secondary metabolites on cancer cells has led to the discovery of major pharmaceutical chemotherapeutics. About 51% of the officially prescribed anticancer drugs are of plant origin. Remarkable examples of plant-derived anticancer agents are Camptothecin and Taxol, isolated by a bioactivity-directed fractionation of the crude plant extracts. These compounds kill cancer cells by unique and previously unknown mechanisms of action, and together they have been approved for treatment of ovarian, breast, lung, and colon cancers and Kaposi’s sarcoma [2].

A key factor in cancer genesis is the process of chronic and repeated inflammation, affecting tissue homeostasis and repair [3]. The functional relationship between inflammation and cancer is not new. In 1863, Virchow suggested the hypothesis that cancer arises at areas of chronic inflammation, based on his presumption that some classes of irritants, together with the tissue injury and the inflammation they cause, enhance cell proliferation [4]. Thus, it has been considered a prospective approach to harness the inflammatory outburst and normalize the host response of the patient which would lead to decreasing the tumor-promoting properties of the infiltrating cells (i.e., pro-inflammatory cytokines) while increasing their tumor-suppressing properties (anti-inflammatory cytokines) [5]. Lipoxygenases are non-heme iron (Fe^2+^)-containing enzymes incorporating molecular oxygen into polyunsaturated fatty acids [6]. LOXs and their catalysis products are associated with carcinogenic processes such as tumor cell proliferation, differentiation, and apoptosis [7]. They are involved in the different aspects of pro- and anti-inflammatory processes in the organism. Several lines of evidence have proven the crucial role of LOXs in cancer. It has also been shown that the 5-LOX metabolite LTB4 is capable of activating the transcription factor NF-κB in cancer cells, which suggests a tumor promoting role via this route [8]. Thus, in recent decades, LOX inhibitors have raised research interest as a potential new class of anticancer agents [9].

In vitro cultivation has been shown to support the successful biosynthesis of compounds with anticancer activity in a number of species [10]. In addition, a number of studies have shown that in vitro cultivation often results in the production of new compounds which are not characteristic for the field-cultivated plant [11,12].

The *Sideritis* genus, belonging to the Lamiaceae family, encompasses over 150 species with distribution through the temperate and tropical areas of the Northern hemisphere [13,14]. *Sideritis scardica* Griseb. (“Olympus tea”, “Pirin Tea”, or “Mursalski Tea”) is a Balkan endemic species. It is a xerophytic herbaceous perennial, growing at high altitudes over 1000 m a.s.l. [15,16]. It is traditionally utilized as a pulmonary treatment, as well as anti-flu and wound-healing remedy [17]. Recent research has brought insight also into other pharmacologically relevant activities of different preparations of the plant. Different types of its extracts have been shown to possess cytotoxic effects on murine melanoma B16, human leukemia HL-60 cells, as well as C6 rat glioma cells, these effects being attributed to reactive oxygen species induction by the chemical constituents present in the studied preparations [18]. The latter work showed cytotoxic effects of *S. scardica* extracts to be at least partially mediated by the flavonoids apigenin and luteolin. Phenolic components in *S. scardica* extracts have been shown by other authors to exhibit dose-dependent anti-inflammatory and gastroprotective activities, as well as cytotoxic activity against cancer cells ([19] and references cited therein). Phenolic compounds found in *Sideritis* species have been systematized in three main types—phenylethanoid glycosides, flavonoid glycosides and phenolic acids [16,20,21,22,23]. Recently, Yücer et al. [24] investigated the anti-inflammatory and anticancer activities of phytochemicals of the *Sideritis* genus. By means of literature survey of available data on the genus representatives, the authors set up a chemical library containing 657 chemical components. The survey outlined overall 32% of phenolics, 33% diterpenes and 35% mono-, sesqui-, and triterpenes, sterols, and straight-chain aliphatic compounds as being characteristic for the secondary metabolite pool of the *Sideritis* genus. Of the phenolic compound pool, the share of flavonoids and their derivatives was 62% and of phenylethanoids and phenylpropanoids, 7%. Compounds were investigated for their capacity of binding to NLRP3 and NF-κB proteins in silico by virtual drug screening and molecular docking. *S. stricta* extracts were also tested in vitro by microscale thermophoresis and phenolic compounds, analyzed by liquid chromatography–high-resolution mass spectrometry. In our previous work, cytotoxicity of commercially obtained *S. scardica* sample on MCF7 cells was obtained [25]. Assessing the cytotoxicity of extracts of in vitro-propagated, ex vitro, and in situ *S. scardica* plants has shown promising results on HeLa (cervical adenocarcinoma), HT-29 (colorectal adenocarcinoma), and MCF-7 (breast cancer) human cancer cell lines [26].

In our previous research, a comparative study of the phenolic profile of wild-collected and in vitro-cultivated *S. scardica* Griseb. outlined the capacity of tissue culture-obtained material to accumulate two phenylethanoids and five flavone glycosides, not detected in the wild-collected plant material. In addition, supplementation of activated charcoal in the culture medium was shown to stimulate flavone glycoside accumulation, leading to an elevated flavone/phenylethanoid ratio, as compared with plant growth regulators treatments [27]. As far as the authors of the present work are aware, there has been no extensive research on prostate cancer and LOX-inhibitory activity of *S. scardica* preparations of wild or in vitro-cultivated material. This motivated us to perform a comparative study of the PC cytotoxic potential and LOX-inhibitory capacity of tissue culture and wild-collected *S. scardica*.

## 2. Results

### 2.1. 15-LOX Inhibition of In Situ- and In Vitro-Obtained Plant Material

**Figure 1:**
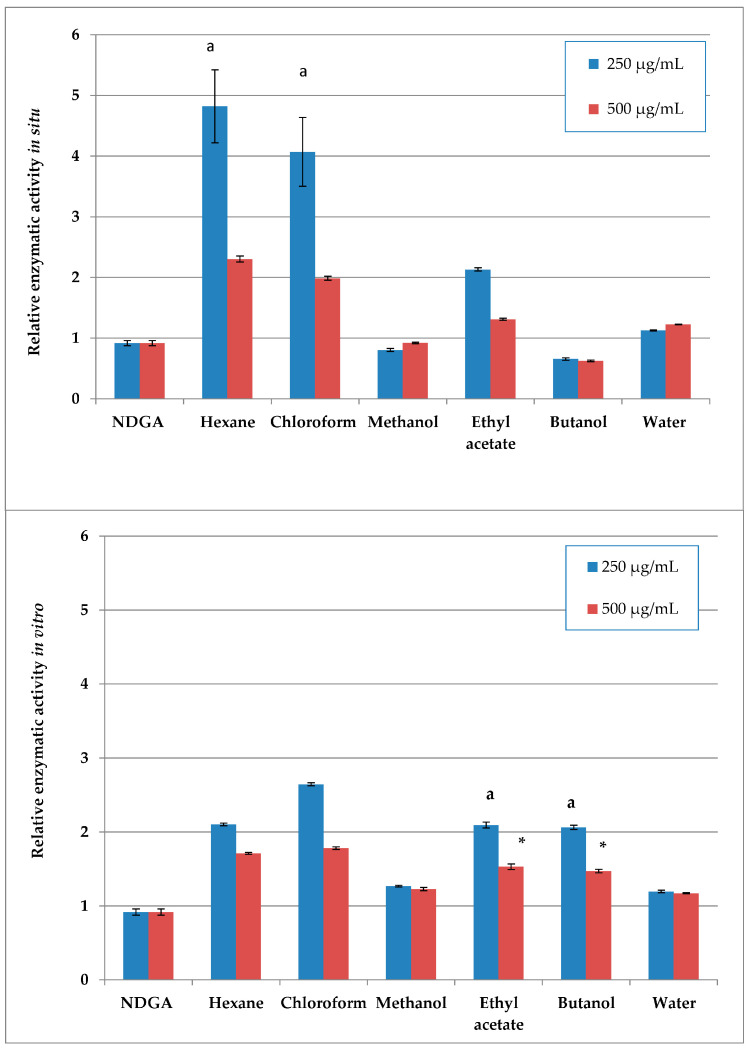
Relative enzymatic activity of the in situ (**up**) and in vitro (**down**) plant preparations [%], calculated as the enzymatic activity of each sample as a percentage of the LOX 100% initial activity; results are normalized to the enzyme activity of the 2.5 µM NDGA inhibited control. *Same letters and asterisk denote statistically insignificant differences when comparing the parameter for one and the same concentration*.

Of the wild-collected samples, the methanol extract and its butanol fraction showed the highest LOX-inhibitory activity, exceeding the NDGA inhibition control, followed by the water fraction with its slightly lower inhibitory activity (Figure 1). Of the in vitro samples, the methanol extract and its water fraction showed the highest activity, although lower than the control.

Higher concentration dependence of the effect was observed for the less active plant preparations (hexane and chloroform, as well as the ethyl acetate in situ and ethyl acetate and butanol in vitro preparations), with significant differences in enzyme inhibition between the two tested concentrations.

### 2.2. Cytotoxicity of In Situ- and In Vitro-Obtained Plant Material

**Figure 2:**
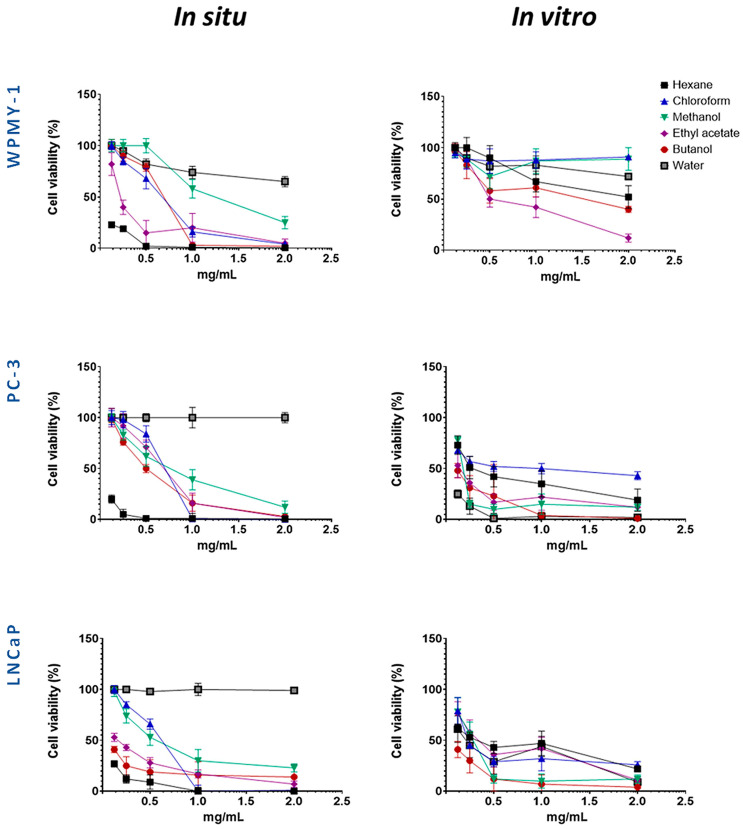
Evaluation of the selective cytotoxic activity of wild-collected and in vitro-cultivated *S. scardica* preparations on the viability of prostate cancer (PC-3 and LNCaP) and normal (WPMY-1) cell lines after 24 h of treatment.

The cytotoxic effects of *S. scardica* extracts and fractions were evaluated on a non-malignant prostate stromal cell line (WPMY-1) and prostate cancer cell lines to determine their potency and selectivity. The cells were exposed to five concentrations of the preparations for 24 h. The corresponding IC_50_ values are presented in Table 1, while the dose–response curves in Figure 2 illustrate the cytotoxic profiles of the extracts and fractions across a wide concentration range.

Several preparations exhibited selective cytotoxicity against both cancer cell lines, as compared to the normal one. These were the methanol extracts from in situ- and in vivo-cultured *S. scardica*, as well as hexane and chloroform extracts, together with butanol and water fractions, from in vitro-cultured *S. scardica*. Notably, the butanol fraction from in situ-collected *S. scardica* was selective for LNCaP cells, while the ethyl acetate fraction from in vitro-cultivated *S. scardica* was selective for PC-3 cells.

**Figure 3:**
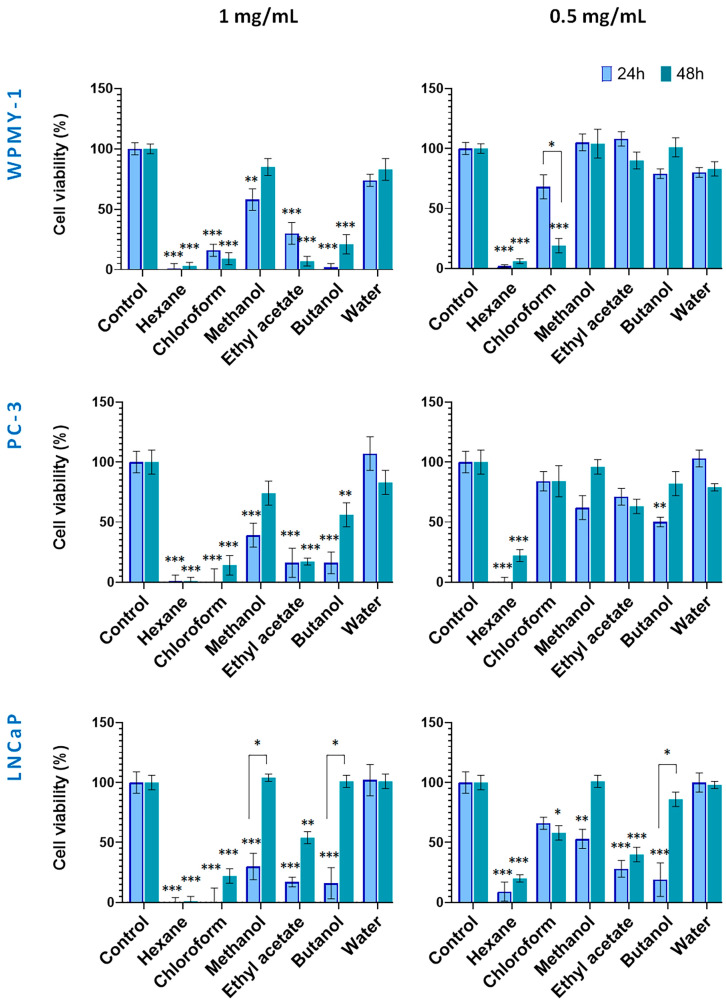
Effect of wild-collected *S. scardica* plant preparations on viability of prostate cancer and normal cells. The viability of cells was evaluated after exposure at concentrations of 0.5 mg/mL and 1 mg/mL for 24 and 48 h. Data are presented as the percentage of absorbance compared to untreated control cells (mean ± SD, n = 3). Significant differences between the control cells and cells treated using two-way ANOVA followed by Tukey’s post hoc test. Statistical significance is indicated as follows: *p* < 0.03 (*), *p* < 0.002 (**), and *p* < 0.001 (***).

**Figure 4:**
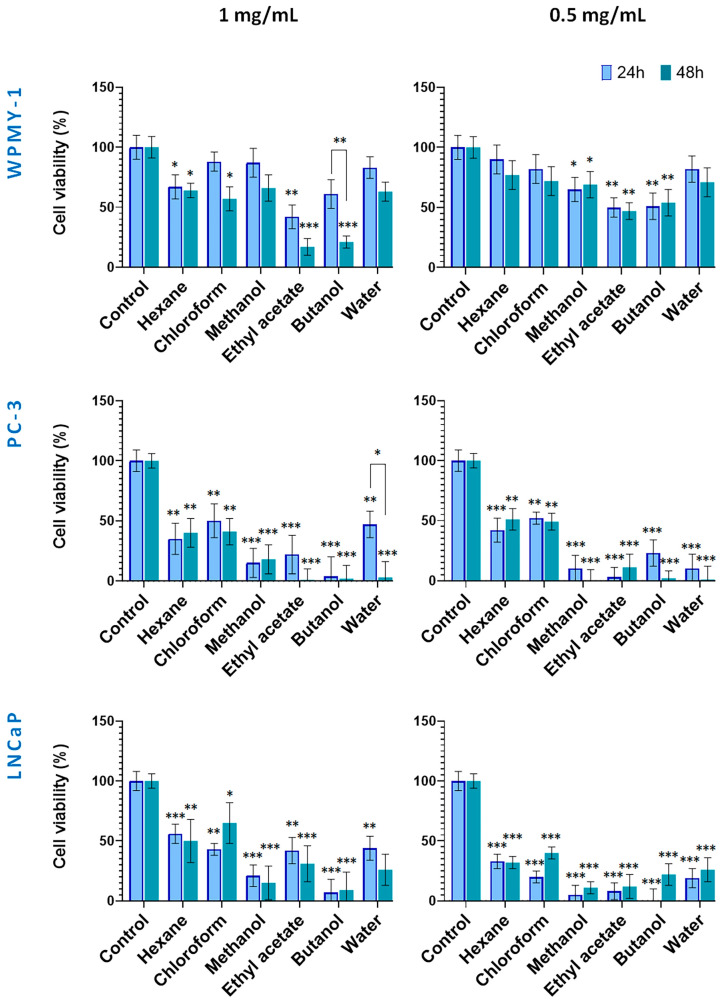
Effect of in vitro-cultivated *S. scardica* plant preparations on viability of prostate cancer and normal cells. The viability of cells was evaluated after exposure at concentrations of 0.5 mg/mL and 1 mg/mL for 24 and 48 h. Data are presented as the percentage of absorbance compared to untreated control cells (mean ± SD, n = 3). Significant differences between the control cells and treated using two-way ANOVA followed by Tukey’s post hoc test. Statistical significance is indicated as follows: *p* < 0.03 (*), *p* < 0.002 (**), and *p* < 0.001 (***).

Based on the IC_50_ values obtained (Table 1), two concentrations (1 mg/mL and 0.5 mg/mL) were selected to evaluate the cytotoxic effects on cancer cell lines while minimizing toxicity to the normal prostate line (WPMY-1), allowing for an accurate assessment of both partial and maximal cytotoxic effects. Experiments were conducted at two time points (24 and 48 h) to capture both early and delayed responses, providing insight into the time-dependent activity and selectivity of the preparations (Figure 3 and Figure 4).

Figure 3 illustrates the cytotoxic effects of preparations from wild-collected *S. scardica*. At 1.0 mg/mL, the hexane and chloroform extracts exhibited strong cytotoxicity toward all cell lines within 24 h. In contrast, the methanol extract and its water fraction exhibited low activity. Cancer cells were also highly sensitive to the ethyl acetate and butanol fractions, with viability reduced to 16–17%. After 48 h, the hexane and chloroform extracts remained the most active (1–22% viability), whereas among the polar preparations, the methanol and butanol fractions displayed decreased cytotoxicity. The ethyl acetate fraction continued to affect WPMY-1, PC-3, and LNCaP cells (7%, 17%, and 54% viability, respectively).

As a general observation, no principal differences in viability were observed between normal and cancer cells when treated with the high concentrations of in situ preparations within both incubation periods.

At 0.5 mg/mL, cytotoxicity displayed higher cell type selectivity. After 24 h, the hexane extract showed strongly toxic to all cell lines. Noteworthy, the WPMY-1 cells demonstrated less sensitivity to methanol extract and its ethyl acetate and butanol fractions as compared to cancer cells. After 48 h, the cytotoxicity of the hexane, methanol extracts, and butanol fractions decreased in cancer cells. Notably, the ethyl acetate fraction at 0.5 mg/mL demonstrated selective and time-stable cytotoxicity against LNCaP cells.

The cytotoxic potential of *S. scardica* extracts derived from in vitro-cultivated plant material is presented in Figure 4. Noteworthy, at both tested concentrations, the normal WPMY-1 cell line displayed a tendency of lower sensitivity to the applied preparations, as compared with the two prostate cancer lines. As a general observation, the polar *Sideritis* preparations had a stronger cytotoxic effect as compared with hexane and chloroform ones. In addition, except for the 1 mg/mL WPMY-1 (all but the hexane treatments), the 1 mg/mL water fraction on PC-3 and the 0.5 mg/mL LNCaP chloroform treatments, prolonging of the incubation period from 24 up to 48 h did not lead to significant restoration of cell vitality.

Thus, in the 1.0 mg/mL 24 h treatment, all extracts and fractions showed moderate to strong cytotoxicity against the PC cell lines, reducing viability down to 4% and 56%. As discussed above, the WPMY-1 cells were more resistant, maintaining high viability (83%) under methanol, chloroform, and water preparation treatments, and a moderate one (from 42 to 67%) under the ethyl acetate, butanol, and hexane fraction treatments.

After 48 h of incubation, a tendency of increasing cell toxicity of all tested preparations (but the hexane one) was established for the normal WPMY-1 cells, this effect being noted also for the water fraction treatment of the PC-3 cells.

In addition, in PC-3 cells, the strong cytotoxic effects of the polar preparations reduced cell viability to 0% for the ethyl acetate, butanol, and water preparations, and to 18% for the methanol extract. Treatments of cell lines with the lower concentration (0.5 mg/mL) of the in vitro *Sideritis* preparations followed a similar pattern as discussed above with the marked effect of lack of restoration of cell viability after 48 h of incubation, except in the case of the chloroform extract on the LNCaP cells.

### 2.3. Phytochemical Profile of Wild-Collected and In Vitro-Obtained Plant Material

**Figure 5:**
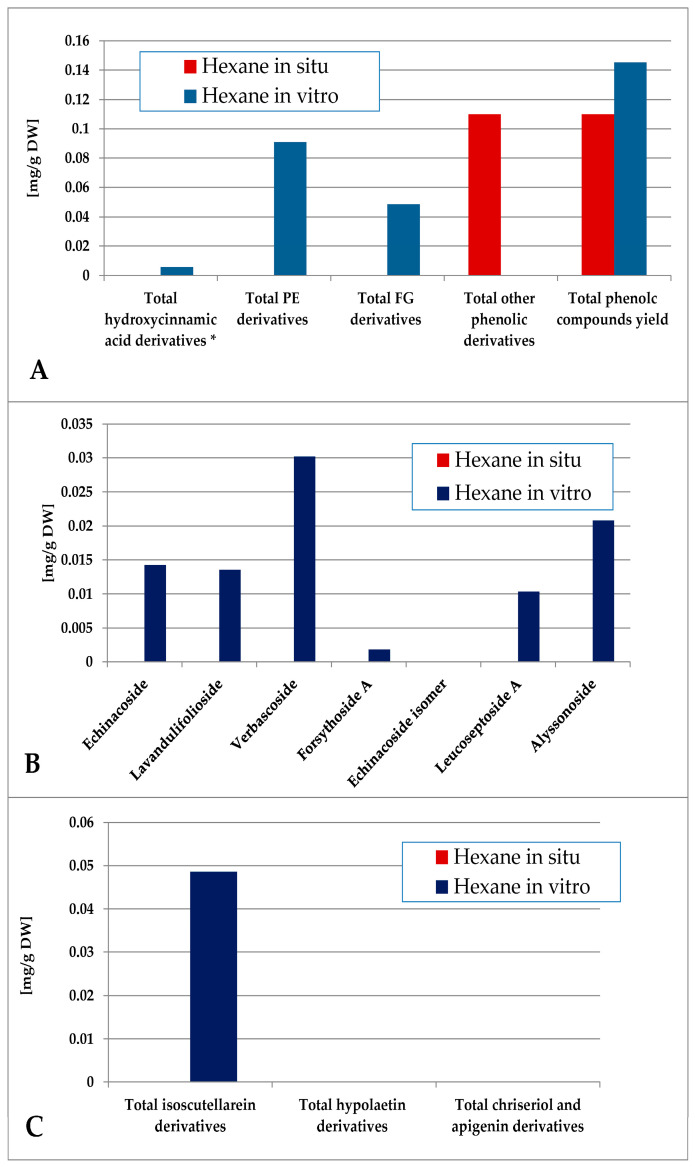
Characterization of polyphenolic profile of in situ and in vitro hexane extract of *S. scardica*. Total levels of analyzed groups (**A**) and content of PE (**B**) and FG (**C**) derivatives. * Only 5-Caffeoylquinic acid forming the hydroxycinnamic pool in situ. *Mean of three measurements; RSD 0.2–3.7%*.

The contents of individual phenolic compounds in the *Sideritis* samples are presented in Table S1 (wild-collected) and Table S2 (in vitro-cultivated). The total phenolic content was calculated as the sum of the individual compound values. Quantitative data represent the mean ± SD of three independent replicates (n = 3). Values reported for certain compounds in the hexane extracts are close to the limit of quantification (LOQ) and should therefore be considered as trace amounts without significant quantitative contribution. Minor variations among samples are expected and can be attributed to natural differences in growth conditions and collection sites. The data were also grouped according to functional classes, including hydroxycinnamic acid derivatives, phenylethanoid glycosides (PE), and flavone glycosides (FG). When comparing the most non-polar hexane extract of the in situ- and in vitro-obtained plant material, logically it was poor in polar compounds, as none of the identified components were established for the in situ material (Figure 5). Though in low amounts, phenylethanoids dominated over flavone glycosides, the most abundant PE components in vitro being verbascoside and alysonoside and isoscutellarein derivatives—the most abundant FG.

**Figure 6:**
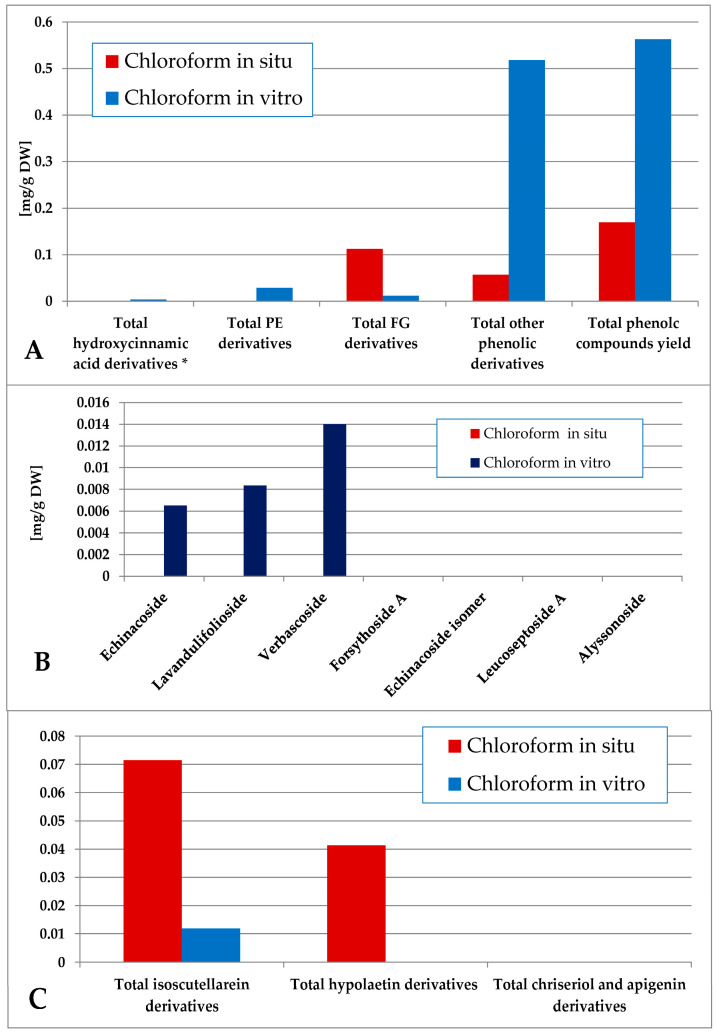
Characterization of polyphenolic profile of in situ and in vitro chloroform extract of *S. scardica*. Total levels of analyzed groups (**A**) and content of PE (**B**) and FG (**C**) derivatives. * Only 5-Caffeoylquinic acid forming the hydroxycinnamic pool in situ. *Mean of three measurements; RSD 0.2–3.7%*.

The content of total phenolic compounds in the chloroform extract was again higher in the in vitro plant material (consisting mainly of other derivatives), although low amounts of phenolic compounds could also be established in situ (Figure 6). The in vitro material yielded mostly PE (with domination of verbascoside) with low amounts of FG (isoscutellarein derivatives), and the in situ material yielded FG (isoscutellarein dominating over hypolaetin derivatives). The hexane and chloroform extracts of the in vitro plant material also yielded traces of the 5-CQA, which, for the in situ plant material, was allocated in the polar preparations (methanol extract and its ethyl acetate, butanol and water fractions, Tables S1 and S2).

**Figure 7:**
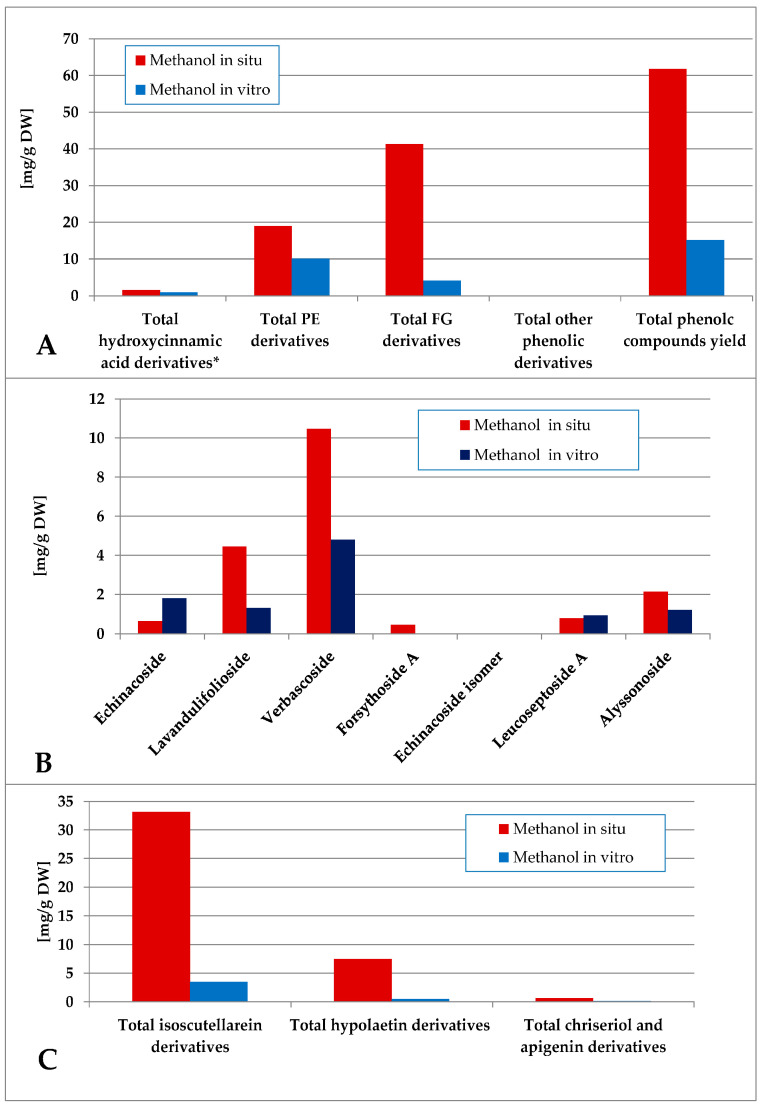
Characterization of polyphenolic profile of in situ and in vitro methanol extract of *S. scardica*. Total levels of analyzed groups (**A**) and content of PE (**B**) and FG (**C**) derivatives. * Only 5-Caffeoylquinic acid forming the hydroxycinnamic pool in situ. *Mean of three measurements; RSD 0.2–3.7%*.

The methanol extraction yielded a more complex polar compound profile of the obtained preparations (Figure 7). Thus, the in situ plant material was significantly more enriched with phenolic compounds, being a mixture of FG and PE, the first group being the dominant one. With significantly lower levels of phenolic components, the PE were the dominant ones in vitro. Verbascoside and lavandulifolioside dominated in the PE pool and isoscutellarein derivatives dominated the FG pool in situ. In vitro, verbascoside was the dominating PE component, much higher than the isocutellarein derivatives which dominated the FG pool.

**Figure 8:**
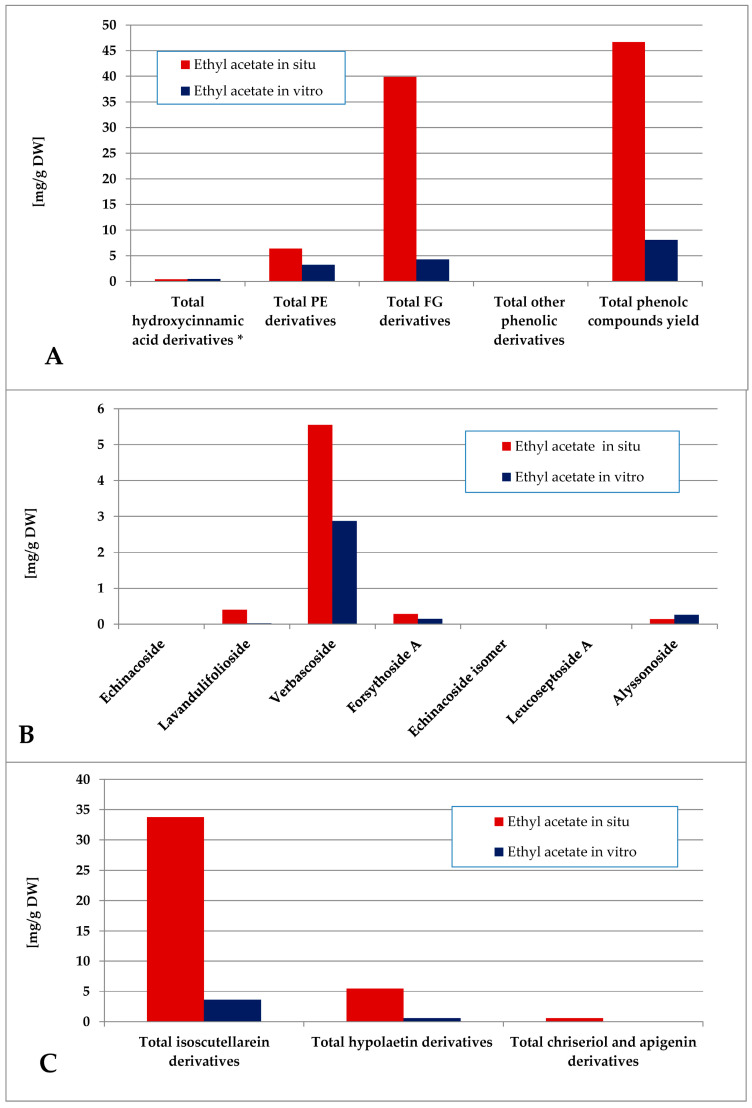
Characterization of polyphenolic profile of in situ and in vitro ethyl acetate fraction of the methanol extract of *S. scardica*. Total levels of analyzed groups (**A**) and content of PE (**B**) and FG (**C**) derivatives. * Only 5-Caffeoylquinic acid form the hydroxycinnamic pool in situ. *Mean of three measurements; RSD 0.2–3.7%*.

The total phenolic content of the ethyl acetate fraction of the wild-collected plant material was almost 6 times higher than the one of the in vitro-obtained one (Figure 8). The dominating polyphenolics in situ were the FG, which represented almost the entire amount of isoscutellarein derivatives. The in vitro-obtained ethyl acetate fraction was characterized by a slight FG over PE prevalence, with verbascoside being almost the only PE component and isoscutellarein derivatives almost entirely constituting the FG pool.

**Figure 9:**
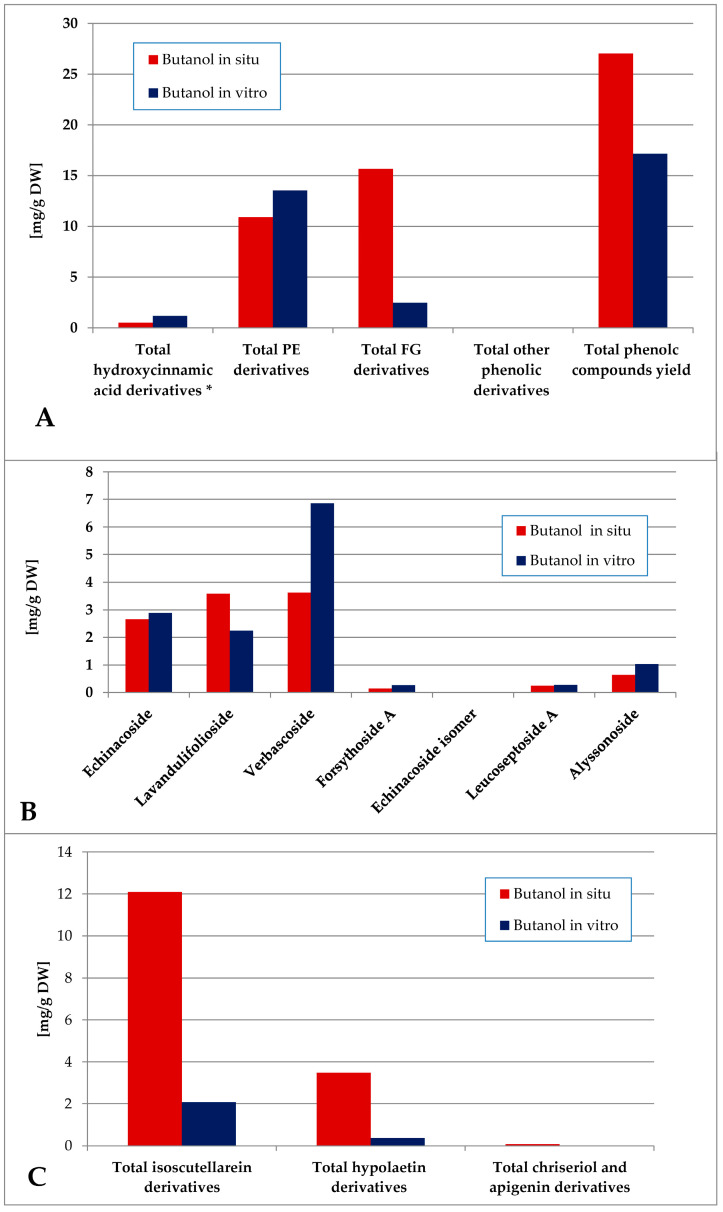
Characterization of polyphenolic profile of in situ and in vitro butanol fraction of the methanol extract of *S. scardica*. Total levels of analyzed groups (**A**) and content of PE (**B**) and FG (**C**) derivatives. * Only 5-Caffeoylquinic acid form the hydroxycinnamic pool in situ. *Mean of three measurements; RSD 0.2–3.7%*.

The butanol fraction of the in situ-obtained plant material was characterized by a higher total phenolics yield as compared with the in vitro one (Figure 9). PE dominated over FG in vitro, and FG dominated in situ. The in vitro levels of verbascoside (the dominant PE) significantly exceeded the in situ levels and isoscutellarein derivatives were the dominating FG both in situ and in vitro.

**Figure 10:**
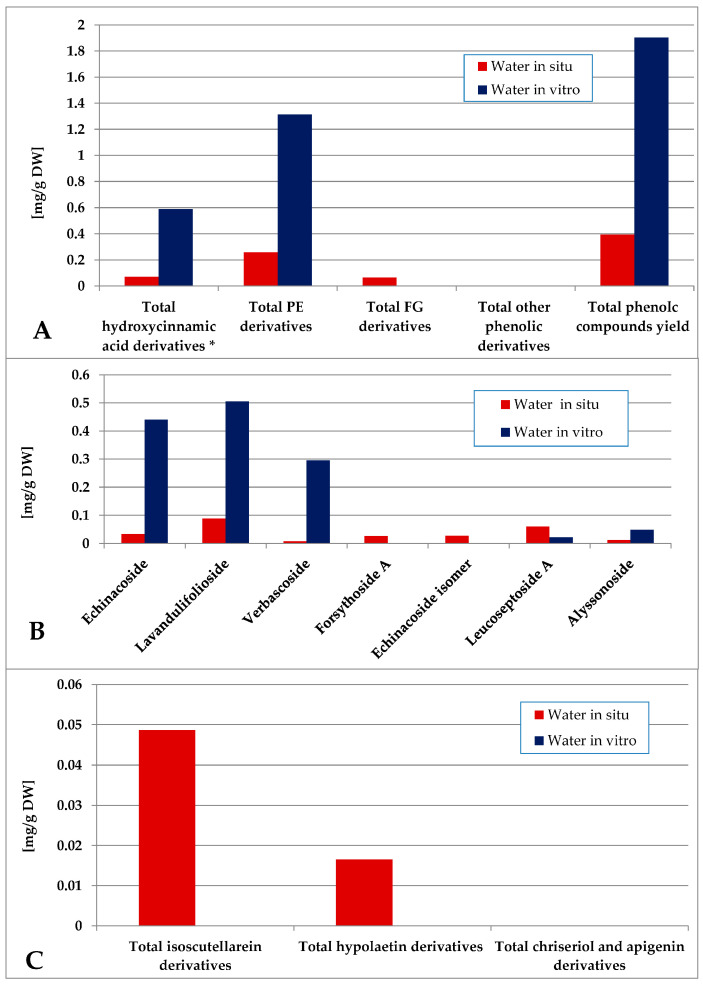
Characterization of polyphenolic profile of in situ and in vitro water fraction of the methanol extract of S. scardica. Total levels of analyzed groups (**A**) and content of PE (**B**) and FG (**C**) derivatives. * Only 5-Caffeoylquinic acid forming the hydroxycinnamic pool in situ. *Mean of three measurements; RSD 0.2–3.7%*.

The water fraction of the in vitro plant material was characterized by a significantly higher total phenolics yield as compared with the in situ plant material (Figure 10, Tables S1 and S2). The in vitro-obtained water fraction contained only PE derivatives and 5-CQA in contrast with the in situ one which contained a mixture of low amounts of PE, FG and 5-QCA. Lavandulifolioside, echinacoside and verbascoside dominated PE in vitro, with low levels of lavandulifolioside and leucoseptoside A in situ. Isoscutellarein derivatives dominated the FG of the in situ water fraction.

**Figure 11:**
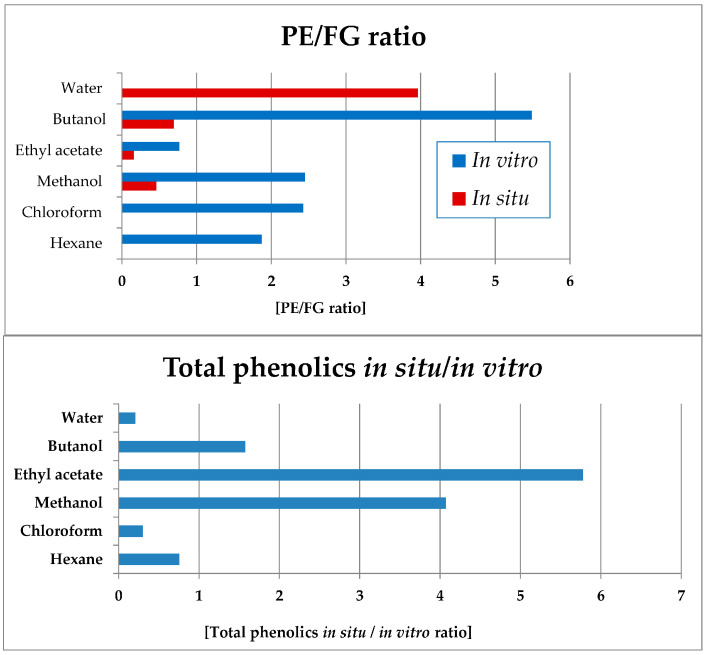
Ratios of total PEs towards FGs (**up**) in vitro and in situ; ratio of total phenolic compounds yields when comparing the in situ and in vitro extracts and fractions of *S. scardica* (bars in blue, **below**). *PE/FR ratio not presented for the in vitro water fraction in which only PE and no FG compounds were present*.

Notably, in all in vitro *Sideritis* preparations, PE significantly dominated over the FG (Figure 11). The water (completely lacking FG) and butanol in vitro fractions were characterized with the highest PE domination in vitro and the ethyl acetate—the lowest values of this parameter. Of the in situ-obtained preparations, the highest PE/FG ratio was established for the water, as well as butanol and methanol (significantly lower) fractions.

Data processing was also performed in order to assess the ratio of the in situ/in vitro total phenolic levels in the obtained plant preparations (Figure 11). Thus, the ethyl acetate, methanol and butanol preparations of the in situ-obtained plant material were considerably more enriched in total phenolic compounds as compared with the respective in vitro-obtained preparations. The water, chloroform, and hexane in vitro preparations were more enriched in phenolic compounds as compared with the in situ preparations.

## 3. Discussion

The sum of total phenolic compound yield in all in situ plant preparations was 136.29 mg/g DW, and 43.05 mg/g DW for all in vitro preparations (Figure 5, Figure 6, Figure 7, Figure 8, Figure 9 and Figure 10, Tables S1 and S2), evidencing the higher potential of the wild-collected material to produce phenolic compounds as compared with the tissue culture of *S. scardica* Griseb.

Compared to previous reports, which often demonstrated higher phenolic accumulation in *Sideritis* species propagated in vitro [12,26], our results revealed lower overall phenolic content. This discrepancy may be due to differences in culture conditions (presence/absence of plant growth regulators), elicitation strategies, or extraction protocols, all of which are known to strongly affect metabolite profiles in tissue cultures [11,27]. However, the present work demonstrated a distinct enrichment in phenylethanoid glycosides (PEs) in the in vitro as compared to in situ plant material (Figure 11). In addition, the approach of producing plant preparations with different polarity in the present work led to obtaining interesting dependencies in the allocation of component types, which in turn led to differential effects on the biological activity potential of the in situ- vs. in vitro-obtained preparations. When comparing the profiles of the non-polar (hexane and chloroform) *Sideritis* preparations, it was established that a higher proportion of polar compounds was allocated to them in vitro as compared to in situ. This observation is logical given the specificity of the in vitro-obtained plant material—a significantly tenderer cell wall, low amounts of cuticular wax, and the almost missing tissue lignification make in vitro-obtained plant material much more easily extractable. This is also evidenced by the higher extraction yield of all in vitro preparations as compared with the in situ ones (Tables S1 and S2). Thus, the mechanical effect of ultrasonic extraction has led to the allocation of some of the polar compounds to the non-polar in vitro extracts. It is however important to mention that terpenoid compounds (especially diterpenes present in flowering aerial parts of in situ plant material), which have not been investigated in the present work, could also affect the obtained dependencies and especially the profound cytotoxic effect of the non-polar in situ preparations of the plant.

The above-discussed results on the qualitative and quantitative composition of the obtained *Sideritis* preparations outlined the ethyl acetate fraction of the in situ material as the highest in phenolic compound accumulation and most FG-dominated of all in situ- and in vitro-obtained samples (Figure 5, Figure 6, Figure 7, Figure 8, Figure 9, Figure 10 and Figure 11). On the contrary, all in vitro material preparations were markedly characterized with a significant domination of PE over FG, disregarding the overall lower phenolic compounds levels accumulated in vitro (Figure 5, Figure 6, Figure 7, Figure 8, Figure 9, Figure 10 and Figure 11).

Extensive research on numerous species has been performed throughout the plant kingdom in the search of bioactive plant preparations or isolated compounds with LOX-inhibitory activity. Among these, as reviewed by Lončarić et al. [28], are representatives of the families Apiaceae, Asteraceae, Clusiaceae, Fabaceae, Lamiaceae, Melastomataceae, Ericaceae, Menispermaceae, Rosaceae, Sapindaceae, etc. The tested bioactive plant preparations have been obtained with solvents of different polarity (hexane, dichloromethane, chloroform, methanol, ethyl acetate, butanol, and ethanol) and from different plant parts (such as blossoms, fruits, leaves, roots, aerial parts, whole plant and stem bark). Phytochemical analyses of bioactive plant preparations with established LOX-inhibition activity have shown the presence of diterpenoids, lignans, flavonoid glycosides and aglycones, proantocyanidins, phenylethanoids, saikosaponins, terpenoids, coumarins, other phenolic compounds, phenolic acids, steroids, etc.

PC is the most common male malignancy and the second leading cause of the cancer-related deaths [29]. Prostate cancer (PC) therapy today includes the application of a complex of approaches including radical prostatectomy, radiation therapy, androgen deprivation (in cases of metastatic development), and chemotherapy [30]. Chemotherapeutic agents most often applied are docetaxel (microtubule assembly inhibitor), as well as its derivative cabazitaxel [31], the topoisomerase inhibitor mitoxantrone [32], and estramustine (topoisomerase inhibitor and microtubule inhibitor) [33]. Most of the PCs are adenocarcinomas with glandular initiation and are characterized by the expression of the AR and PSA markers [34]. In vitro model systems, utilized for screening the cytotoxicity of potential chemotherapeutic agents for PC, include the two models investigated in the present work. The epithelial PC-3 is a grade IV adenocarcinoma cell line, isolated from bone metastasis of PC. These cells do not express AR and PSA and they possess an androgen-independent proliferation, making hormonal therapy ineffective. PC-3 cells are highly aggressive, unlike most clinical cases of PCs [34]. On the contrary, the LNCaP line (derived from lymph node metastasis) is AR- and PSA-expressing, being an androgen-dependent adenocarcinoma, with clinical behavior in most of the encountered PC cases [34]. Research has provided evidence of PC-3 cells expressing features of prostatic SCNCs [34]. Regarding prostate cancer cell cytotoxicity, higher potential in the present work was established for the FG-enriched fractions. A limitation of the present study is the absence of a conventional drug control, such as docetaxel, which is the first-line chemotherapeutic for advanced prostate cancer. Literature values indicate that docetaxel achieves cytotoxic effects in PC-3 and LNCaP cells at nanomolar concentrations (2–10 nM) [35], while the *S. scardica* preparations tested here required much higher concentrations. Thus, our findings should be viewed in the context of preliminary screening, highlighting selectivity trends rather than pharmacological potency. Moreover, our findings confirm the above-discussed results of Jeremic et al. [18] on the cytotoxicity of *S. scardica* plant preparations, established against rat glioma C6 line, attributed to the luteolin and apigenin present in the plant extracts. Future work will include direct benchmarking of extracts and fractions against docetaxel and other clinically relevant drugs to better evaluate translational potential of obtained tendencies. Numerous plant species have been investigated for their cytotoxic activity on cancer cell lines within the research efforts to identify bioactive preparations with the capacity to inhibit PC cell growth. An extensive review by Thomas-Charles and Fennel [30] reveals the potential of secondary metabolites of diverse chemical types, which have been found to exhibit cytotoxicity in different PC cell lines. The species are members of the Juglandaceae, Crassulaceae, Moraceae, Fabaceae, Salicaceae, Malpighiaceae, Euphorbiaceae, Meliaceae, Rutaceae, Bixaceae, Malvaceae, Brassicaceae, Lythraceae, Geraniaceae, Lamiaceae, Boraginaceae, Apocynaceae, Asteraceae, Apiaceae, Sapotaceae, Dicksoniaceae, Nephrolepidaceae, Dryopteridaceae, with terpenes, sesquiterpenes, sesquiterpene lactones, naphthoquinones, phenolics, flavonoids, and alkaloids identified in the bioactive plant preparations obtained from them. As discussed above, in their recent work, Yücer et al. [24] clarified anti-inflammatory and anticancer mechanism features of *Sideritis* sp. bioactive compounds by virtual screening and molecular docking. Thus, two biologically active compounds—verbascoside (PE) and apigenin 7,4′-bis(trans-*p*-coumarate) (acylflavone)—were identified as the ones with the highest binding affinity towards inflammation-related NLRP3 and NF-κB proteins, with the lower Kd values (higher affinity) being verbascoside for both proteins. Subsequent in vitro experiments provided favorable results for the anticancer activity of verbascoside towards 49 tumor cell lines.

**Figure 12:**
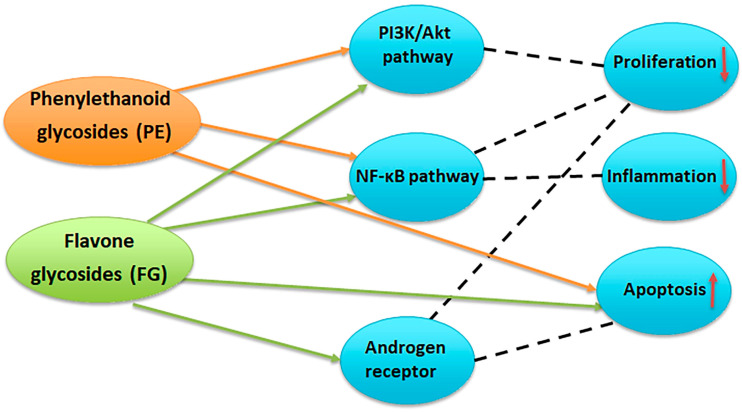
Potential targets of phenylethanoid glycosides (PE) and flavone glycosides (FG) in prostate cancer. PE inhibit the PI3K/Akt and NF-κB pathways, leading to reduced cell proliferation, decreased inflammation, and induction of apoptosis. FG modulate PI3K/Akt, NF-κB, and androgen receptor signaling, resulting in suppression of proliferation and inflammation, enhanced apoptosis, and interference with androgen-driven tumor growth [24,36,37,38,39,40].

**Figure 13:**
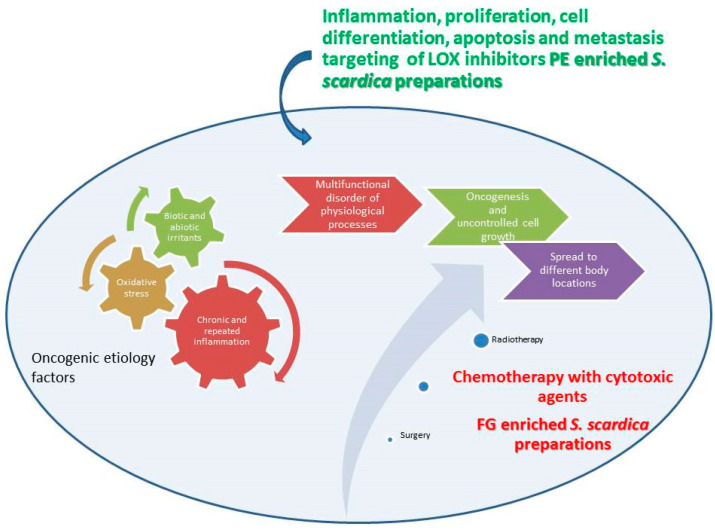
Schematic representation of possible roles of PE and FG LOX inhibitors and PC cell cytotoxic agents. The Figure illustrates references [1,2,3,4,5,6,7,8,9].

A bibliographic survey on the effects of compounds with PE and FG structure on proliferation, inflammation, and apoptosis (Figure 12) has shown the overlapping and interrelating roles of the representatives of the two chemical groups. Interestingly, when summarizing the above bibliographical data, the results of the present work seem to be indicative of the possibility of a preferential LOX inhibition of PE-enriched *Sideritis* preparations and direct cytotoxicity of FG-enriched preparations, outlining the potential of PE-enriched preparations to target rather the LOX-related events of oncogenic etiology and FG-enriched preparations and thus exhibit higher relevance as direct cytotoxic agents (Figure 13).

A limitation of this study is that the biological effects observed were not confirmed using purified standards of the identified compounds. Although correlations between phenylethanoid-rich fractions and LOX inhibition, and between flavone glycoside–rich fractions and cytotoxicity, are consistent with literature reports, direct testing of pure standards (e.g., verbascoside, echinacoside, apigenin and luteolin glycosides) is required to clearly establish causality. Future studies will therefore focus on evaluating these compounds individually, either as isolated fractions or commercial standards, to validate their specific roles and to better understand potential synergistic interactions within the complex extract matrix.

This experimental design of the present work was intended to identify relative trends, selectivity, and the potential contribution of different phenolic profiles, rather than to provide precise pharmacological potency values. Future studies will extend these observations by applying full concentration–response analyses of the most active fractions.

Beyond their in vitro relevance, the activities observed suggest a possible translational potential of *S. scardica* preparations as sources of compounds targeting both inflammation and cancer pathways. To advance towards clinical application, further studies including in vivo efficacy models, pharmacokinetic and toxicological evaluation are required. Importantly, the use of in vitro plant culture provides an innovative route for sustainable production of bioactive metabolites. This not only reduces harvesting pressure on endangered wild populations but also offers advantages such as standardized phytochemical composition, reproducibility, and scalability of biomass production under controlled conditions.

As discussed above, with respect to biological activity, earlier studies have reported the cytotoxicity of *S. scardica* extracts in breast (MCF-7), colorectal (HT-29), and glioma (C6) models [18,25,26]. Cytotoxicity of *S. scardica* plant preparations, established against rat glioma C6 line, was attributed to the flavonoid glycosides luteolin and apigenin present in the plant extracts [18]. Our findings extend this knowledge by demonstrating selective effects in prostate cancer cells (PC-3 and LNCaP), which have not been previously addressed. Importantly, the stronger cytotoxicity of flavone glycoside (FG)-enriched fractions observed here aligns with earlier work implicating apigenin and luteolin glycosides in ROS-mediated apoptosis and cell-cycle arrest [19,30], while highlighting their potential translational relevance in prostate cancer therapy.

In contrast, LOX-inhibitory activity was predominantly associated with PE-rich fractions in our study, whereas most reports emphasize flavonoids as major LOX inhibitors [28]. This divergence may reflect structural differences influencing enzyme binding, as verbascoside and related PEs have recently been shown to interact with NF-κB and NLRP3 inflammasome targets [24], suggesting that their role in inflammation-related cancer pathways may be broader than previously recognized. Thus, our comparative approach adds new mechanistic insight by linking phenylethanoid enrichment to LOX inhibition and flavone glycoside enrichment to cytotoxicity.

These distinctions underscore the novelty of our findings and highlight the need for future mechanistic studies, including gene and protein expression analyses, to reconcile discrepancies with previous reports and clarify the translational potential of *S. scardica* bioactive compounds.

From a translational standpoint, the present findings suggest that *S. scardica* preparations may hold promise as sources of lead compounds for drug development targeting inflammation- and cancer-related pathways. Phenylethanoid glycosides, exemplified by verbascoside, could be considered candidates for anti-inflammatory or chemopreventive agents through LOX inhibition and modulation of NF-κB/NLRP3 signaling, whereas flavone glycosides such as luteolin and apigenin derivatives are established inducers of apoptosis and may serve as scaffolds for anticancer therapeutics. Nevertheless, these prospects remain preliminary, as our experiments were limited to in vitro assays. Further translational steps will require testing in in vivo models to define bioavailability, metabolism, and toxicity profiles, followed by dose–response evaluations to determine effective and safe dosage ranges. Such studies are essential before clinical applications or nutraceutical development can be considered.

## 4. Materials and Methods

### 4.1. Plant Material and Tissue Culture Conditions

**Figure 14:**
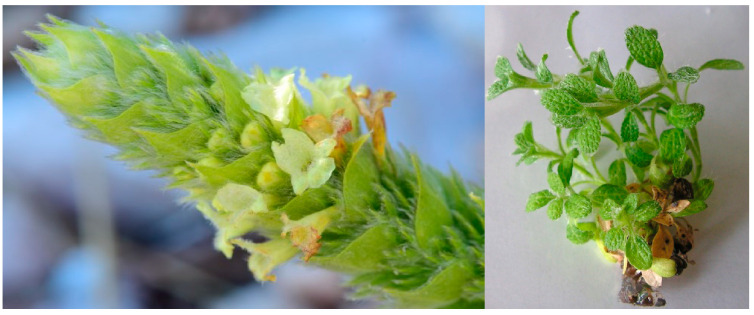
Plant material used in the experiment: wild growing at Schabran peak, Slavyanka Mountain, Bulgaria, (**left**, photo courtesy Ina Aneva) and in vitro-cultivated in the PGR-free medium (**right**) *S. scardica* Griseb.

Wild-growing plant material in the flowering stage as well as seeds for in vitro culture development were collected at the Shabran peak, Slavyanka Mountain, Bulgaria (Figure 14). Stock shoot cultures of the plant were initiated from sterilized wild-collected seeds of the plant as previously described [27]. In vitro-cultivated plant material for the analyses was cultivated in PGR-free medium supplemented with the Murashige and Skoog [41] salts and Gamborg [42] vitamins, 2 mg/L glycine, 30 g/L sucrose and solidified with 6.0 g/L agar (culture media were in-lab prepared by stock-solutions prepared from salts and chemicals, Duchefa Biochemie B.V, Haarlem, The Netherlands). Shoots were cultivated at 25 ± 1 °C at 16/8 h photoperiod for an 8-week period (Figure 14).

The in vitro-obtained plant material was collected and air-dried in a dessicator until constant weight.

### 4.2. Preparation of In Situ and In Vitro Sideritis Plant Samples

Based on the diverse content of biologically active compounds with variable polarity characteristic for the *Sideritis* genus, our aim was to obtain extracts and fractions in which chemical constituents will be differentially distributed. Extraction was aimed to initially defat plant material and then fractionate the methanol extract, rich in a mixture of polar compounds into three separate fractions. Air-dried plant material of the wild-collected and in vitro-cultivated *S. scardica* Griseb. was extracted in triplicate in ultrasonic bath for 10 min at room temperature in the following steps: consecutive hexane, chloroform, and methanol extracts were obtained, followed by liquid/liquid fractionation of the methanol extract to obtain its ethyl acetate, n-butanol, and water fractions. For each of the three extractions with each of the solvents, the ratio of plant material to solvent was 20 mL solvent per gram of DW.

Extracts were evaporated till dry in a rotary vacuum evaporator and kept at −18 °C prior to analyses.

### 4.3. 15-LOX-Inhibition Assays of Plant Samples

Analyses were performed by means of the Lipoxygenase Inhibitor Screening Assay Kit, Cayman Chemical, Ann Arbor, MI, USA. Arachidonic acid was used as a substrate, Nordihydroguaiaretic acid (2.5 µM, IC_70_ at our experimental conditions) as the inhibition control, and 15-LOX standard as positive control. The absorption of the following samples was measured: optical blank of the assay—consisting of the kit assay buffer; positive control sample—15-LOX reference suspended in the in assay buffer; *100%* initial activity sample—15-LOX suspended in assay solvent (DMSO); inhibitor sample—15-LOX and tested plant preparation samples suspended into the assay solvent (DMSO). After incubation, a reaction was initiated with the addition of the substrate (arachidonic acid) and kit chromogen reagent. Absorbance was measured at 490–500 nm. The inhibition percentage of each inhibitor was assayed according to the kit protocol and determined using the following formula:

% Inhibition = [(IA − Inhibitor)/IA] × 100

where IA—100% initial activity sample;
Inhibitor—absorbance of the inhibitor sample.

Results were processed and presented as the relative enzyme activity, calculated as the LOX activity upon application of each of the tested inhibitors, as percentage of the 100% initial activity sample of LOX. Values are normalized to the 2.5 µM Nordihydroguaiaretic acid inhibition (IC_70_ at our experimental conditions) accepted as a reference unit. Plant preparations were tested at concentrations of 250 and 500 µg/mL. Treatment concentrations were selected based on a literature survey of similar works performed by other authors [28]. In order to select the most potent of the preparations in the two tested concentrations, we compared their activity with the one of the NDGA concentrations exhibiting IC_70_ in our experimental conditions.

### 4.4. Cytotoxicity Assays of Plant Samples

The cytotoxic activity of *S. scardica* extracts and fractions was evaluated using two human prostate cancer cell lines, PC-3 (epithelial adenocarcinoma, Grade IV) and LNCaP (epithelial carcinoma), as well as a normal human prostate stromal cell line, WPMY (epithelial fibroblast/myofibroblast). All cell lines were obtained from the American Type Culture Collection (ATCC, Manassas, VA, USA). PC-3 and WPMY cells were cultured in low-glucose DMEM supplemented with 10% and 5% FBS, respectively. LNCaP cells were maintained in RPMI-1640 medium supplemented with 10% FBS.

The cytotoxic effects were assessed using the MTT assay. Cells were seeded in 96-well plates at a density of 2 × 10^4^ cells/well in 100 μL of the appropriate culture medium. After 24 h of incubation, test samples were added. Immediately before use, the dried extracts and fractions were initially dissolved in DMSO at a stock concentration of 200 mg/mL and then diluted in culture medium to the required working concentrations. To minimize any potential solvent effects, the final concentration of DMSO in all wells was maintained below 1%.

Cell viability was determined by adding 10 μL of MTT solution (5 mg/mL) to each well. Cells were then incubated for 3 h at 37 °C in a humidified atmosphere containing 5% CO_2_. Viable cells reduced the yellow MTT salt to insoluble purple formazan crystals. To solubilize the crystals, 100 μL of the solubilization solution (40% dimethylformamide and 16% sodium dodecyl sulfate in 2% glacial acetic acid, pH 4.7) was added to each well, and the plates were then incubated for an additional 20 min at 37 °C with shaking. Absorbance was measured at 544 nm using a FLUOstar OPTIMA microplate reader (BMG Labtech, Ortenberg, Germany). Data from three independent experiments in triplicate were expressed as a percentage of the absorbance relative to untreated control cells (mean of treated wells/mean of untreated control wells) × 100%. DMSO was used as a negative control. The half-maximal inhibitory concentration (IC50) of each *S. scardica* extract and fraction was determined using concentrations ranging from 0.125 to 2 mg/mL. The cells were exposed to the plant preparations for 24 h, and the IC50 values were calculated using GraphPad Prism 8 (Table 1, Figure 2).

### 4.5. Phytochemical Characterization of Plant Samples

Chromatographic separation was carried out on a C18 Eclipse column (150 × 4.6 mm, 5 µm; Agilent, Santa Clara, CA, USA) using a mobile phase composed of 1% formic acid in water (A) and 1% formic acid in methanol (B). A linear gradient was applied: 35% B at 5 min, increasing to 50% at 30 min, reaching 100% at 35 min, and held until 55 min. The flow rate was 0.4 mL/min, and the injection volume was 20 µL.

The HPLC system (Agilent 1100 Series) with diode array detector and coupled to an ion trap mass spectrometer was used for analysis. UV spectra were recorded between 190 and 600 nm and mass detection was performed in negative electrospray ionization (ESI) mode, using nitrogen as the nebulizing gas (65 psi, 12 L/min), with a capillary temperature of 325 °C and ionization voltage of 4 kV. Full scan data were collected over *m*/*z* 100–1200.

Phenolic compounds were identified based on retention times, UV-Vis spectra and mass spectra compared with authentic standards and literature data [16,20,22,43].

Reference standards of 5-caffeoylquinic acid, *p*-coumaric acid, verbascoside and apigenin 7-*O*-glucoside of analytical grade were purchased from Sigma Aldrich (St. Louis, MO, USA), hypolaetin 7-*O*-[6‴-*O*-acetyl]-allosyl(1 → 2)glucoside, 4′-*O*-methylhypolaetin 7-*O*-[6‴-*O*-acetyl]-allosyl(1 → 2)glucoside, 4′-*O*-methylhypolaetin 7-*O*-[6‴-*O*-acetyl]-allosyl-(1 → 2)-[6″-*O*-acetyl]-glucoside, isoscutellarein 7-*O*-[6‴-*O*-acetyl]-allosyl(1 → 2)glucoside, isoscutellarein 7-*O*-[6‴-O-acetyl]-allosyl-(1 → 2)-[6″-*O*-acetyl]-glucoside and 4′-*O*-methylisoscutellarein 7-*O*-[6‴-*O*-acetyl]-allosyl(1 → 2)glucoside, lavandofulioside and alyssonoside were previously isolated from *Sideritis* species and identified in the Laboratory for chemistry of natural compounds at the Institute of organic chemistry with Centre of phytochemistry, Bulgarian Academy of Sciences, Sofia, Bulgaria using NMR (^1^H and ^13^C). Reference standards were isolated in 2023 from a cultivated sample of *S. scardica* collected in 2022 on Pirin mountain, Bulgaria. The isolation and purification of all reference standards were in accordance with previous work [44,45,46]. All compounds were identified by ^1^H and ^13^C Bruker AV 600 (Bruker BioSpin GmbH, Rheinstetten, Germany) in DMSO-d4. The purity of every reference standard was checked by NMR, and values between 96 and 98% were obtained. The stability of standards was followed by HPLC, and it was found that they are stable as solid substances within 2 years, and their methanol solutions were stable within 2 months stored at 4 C.

Quantitative analysis was performed based on chromatograms obtained with a UV–Vis detector. Regarding the selection of the most suitable standard substances for flavonoid quantification, the nature of the aglycone is essential in determining the absorbance properties used in UV detection [47]. Therefore, various derivatives were quantified using an available standard containing the same aglycone, as these compounds exhibit similar UV spectra. Likewise, phenylethanoid derivatives, which share comparable UV absorption characteristics, were quantified as equivalents of the corresponding available standards. In this context, hydroxycinnamic acids were quantified using 5-caffeoylquinic acid as a reference standard at 330 nm, phenylethanoid glycosides as verbascoside equivalents at 330 nm, hypolaetin glycosides with 4′-*O*-methylhypolaetin 7-*O*-[6‴-*O*-acetyl]-allosyl(1 → 2)glucoside at 290 nm, and isoscutellarein as 4′-*O*-methylisoscutellarein 7-*O*-[6‴-*O*-acetyl]-allosyl(1 → 2)glucoside equivalents at 300 nm and chryseriol and apigenin derivatives as apigenin 7-*O*-glucosides at 300 nm.

The method used for quantitative analysis was previously statistically evaluated by calculating the regression equation, correlation coefficient (*R*^2^), linear range, limit of detection (LOD), and limit of quantification (LOQ) for the available standards. The standard curves were established in the range of 1–300 µmol/L. All calculated parameters are presented in Table S3 of the Supplementary Materials.

### 4.6. Statistical Analysis

Statistical analysis of the data of the LOX-inhibition assay were performed by use of Excel 2017 for calculations of means, standard deviation, and standard error of the means. Statistical significances presented in figures are standard error of the mean (SEM) of three measurements. The significance of differences between the treatments for one and the same parameter was analyzed at *p* ≤ 0.05 by a post hoc Tukey-HDS test after performing ANOVA single-factor analysis with the help of the Real Statistics Resource Pack on Excel 2019. For the phytochemical results, the mean of three measurements, RSD 0.2–3.7%, is presented.

Results on cytotoxicity assays are presented as the mean ± standard deviation (SD) from three independent experiments, analyzed using GraphPad Prism version 8. Two-way ANOVA followed by Tukey’s post hoc test was performed to assess cell viability, with statistical significance evaluated at a 95% confidence level.

## 5. Conclusions

The present work discusses for the first time the results of a comparative investigation of LOX-inhibitory activity and PC cytotoxicity of extracts and fractions of wild-collected and in vitro-cultivated *S. scardica* Griseb. The observed differences between PE- and FG-enriched preparations may reflect distinct molecular modes of action. Verbascoside and related phenylethanoids in the literature have been shown to inhibit NF-κB activation and NLRP3 inflammasome signaling, thereby attenuating inflammatory pathways, and to suppress LOX activity by direct enzyme binding. These effects are consistent with the stronger LOX-inhibitory activity observed for PE-dominated fractions in this study. On the other hand, flavone glycosides such as apigenin and luteolin derivatives are widely reported by other authors to induce ROS-mediated apoptosis, inhibit topoisomerase activity, and modulate cell-cycle regulators (e.g., cyclins, CDKs), leading to selective cytotoxicity against cancer cells. Such mechanistic distinctions support our finding that FG-enriched fractions displayed higher prostate cancer cytotoxicity. Future work to shed more light on the trends outlined in the current work should include molecular analyses to validate these pathways in the context of *S. scardica* extracts.

In addition to providing new phytochemical and biological insights, this study highlights the translational potential of *S. scardica* as a sustainable source of bioactive compounds. The selective cytotoxicity of flavone glycoside-enriched fractions and the LOX-inhibitory activity of phenylethanoid-enriched fractions underscore possible applications in prostate cancer and inflammation-related conditions. However, rigorous in vivo studies addressing safety, pharmacokinetics, and dosage optimization will be required to move these findings toward clinical application.

## Figures and Tables

**Table 1:** Cytotoxic activity of *S. scardica* plant preparations against normal prostate stromal cells (WPMY-1) and prostate cancer cell lines (PC-3 and LNCaP) after 24 h treatment.

*S. scardica* Cultures	Extracts/Fractions	IC50 (MTT) (Mean ± SD, mg/mL)		
		WPMY-1 (Normal)	PC-3 (Cancer)	LNCaP (Cancer)
In situ	Hexane extract	<0.125	<0.125	<0.125
	Chloroform extract	0.644 ± 0.19	0.695 ± 0.13	0.598 ± 0.11
	Methanol extract	1.130 ± 0.32	0.702 ± 0.21 ***	0.512 ± 0.16 ***
	Ethyl acetate fraction	0.232 ± 0.08	0.676 ± 0.15 **	0.162 ± 0.04
	Butanol fraction	0.672 ± 0.16	0.484 ± 0.09	<0.125 ***
	Water fraction	na	na	na
In vitro	Hexane extract	1.708 ± 0.6	0.312 ± 0.05 ***	0.290 ± 0.09 ***
	Chloroform extract	na	0.551 ± 0.03 ***	0.256 ± 0.08 ***
	Methanol extract	na	0.187 ± 0.06 ***	0.254 ± 0.08 ***
	Ethyl acetate fraction	0.579 ± 0.22	0.147 ± 0.02 **	0.304 ± 0.06
	Butanol fraction	1.693 ± 0.5	<0.125 ***	<0.125 ***
	Water fraction	na	<0.125 ***	0.204 ± 0.07 ***

na—no activity. Values are expressed as mean ± SD from three independent experiments. Statistical significance between normal and cancer cell lines was assessed using two-way ANOVA with Dunnett’s post hoc test. Statistical significance is indicated as follows: *p* < 0.002 (**), and *p* < 0.001 (***).

## Data Availability

Data are available from the corresponding co-authors upon request.

## Supplementary Materials

The following supporting information can be downloaded at: https://www.mdpi.com/article/10.3390/plants14213263/s1, Table S1: Individual phenolic components in wild-collected *S. scardica* preparations; Table S2: Individual phenolic components in in vitro-cultivated *S. scardica* preparations; Table S3: Regression equation, correlation coefficient (*R*^2^), linear range, limit of detection (LOD), and limit of quantification (LOQ) for the available standards; Figure S1: (a) Chromatograms of various extracts from wild growing *S. scardica* at 330 nm; (b) Chromatograms of various extracts from in vitro-cultivated *S. scardica* at 330 nm.

## Abbreviations

The following abbreviations are used in this manuscript:

AKBA	3-acetyl-11-keto-beta-boswellic acid
AR	Androgen receptor
5-CQA	5-Caffeoylquinic acid
DMEM	Dulbecco’s Modified Eagle Medium
DMSO	Dimethyl sulfoxide
DW	Dry weight
FBS	Fetal bovine serum
FG	Flavone glycoside
FW	Fresh weight
LDL	Low-density lipoprotein
LOX	Lipoxygenase
LT	Leukotriene
MTT	3-[4,5-dimethylthiazol-2-yl]-2,5-diphenyltetrazolium bromide
NDGA	Nordihydroguaiaretic acid
NF-κB	Nuclear factor kappa-light-chain-enhancer of activated B cell
NLRP3	NLR family pyrin domain containing 3
PC	Prostate cancer
PSA	Prostate-specific antigen
PEs	Phenylethanoides
PGR	Plant growth regulator
PUFA	Polyunsaturated fatty acid
RPMI	Roswell Park Memorial Institute
ROS	Reactive oxygen species
SCNC	Small cell (neuroendocrine) carcinoma
