# Supplementary material for: Lipoxygenase Inhibitory Activity and Prostate Cancer Cytotoxicity of In Situ- and In Vitro-Cultivated Balkan Endemic Sideritis scardica Griseb
Source: Plants (Basel). 2025 Oct 25;14(21):3263. doi: 10.3390/plants14213263 (PMC12609379; doi:10.3390/plants14213263)
Supplement: Supplementary file 1 [file plants-14-03263-s001.zip › plants-3843638-supplementary.pdf]

Table S1. Individual phenolic components in wild collected *S. scardica* preparations

S. scardica preparations<br>Extraction yield in total 18.71 %	**Hexane [mg/g DW] 1.33 %**	**Chloroform [mg/g DW] 2.01 %**	**Methanol [mg/g DW] 11.34 %**	**Ethyl acetate [mg/g DW] 0.78 %**	**Butanol [md/g DW] 1.49 %**	**Water [mg/g DW] 1.76 %**
**Hydroxycinnamic acid derivatives**						
5-Caffeoylquinic acid*	nd	nd	1.5635	0.4226	0.5102	0.0706
Caffeic acid derivative	nd	nd	nd	nd	nd	nd
p-Coumaric acid*	nd	nd	nd	nd	nd	nd
**Phenylethanoides**						
Echinacoside	nd	nd	0.6594	nd	2.6631	0.0336
Lavandulifolioside*	nd	nd	4.4509	0.4008	3.5814	0.0894
Verbascoside*	nd	nd	10.4690	5.5533	3.6196	0.0077
Forsythoside A	nd	nd	0.4563	0.2831	0.1412	0.0270
Echinacoside isomer	nd	nd	nd	nd	nd	0.0277
Leucoseptoside A	nd	nd	0.8009	nd	0.2442	0.0607
Alyssonoside*	nd	nd	2.1478	0.1434	0.6460	0.0125
**Isoscutellarein derivatives**						
Isoscutellarein 7-*O*-allosyl(1→2)glucoside	nd	nd	3.5455	0.8687	1.7594	0.0057
Isoscutellarein 7-*O*-[6'''-*O*-acetyl]-allosyl(1→2)glucoside*	nd	0.0374	17.0565	14.6970	8.0828	0.0399
4'-*O*-Methylisoscutellarein 7-*O*-allosyl(1→2)glucoside	nd	nd	0.1886	nd	0.1132	nd
Isoscutellarein 7-*O*-allosyl(1→2)-[6''-*O*-acetyl]-glucoside	nd	nd	0.8061	1.0455	0.2051	nd
4'-*O*-Methylisoscutellarein 7-*O*-allosyl(1→2)-[6''-*O*-acetyl]-glucoside	nd	nd	0.1693	nd	0.0408	nd
4'-*O*-Methylisoscutellarein 7-*O*-[6'''-*O*-	nd	0.0091	2.8844	3.2658	0.7794	0.0017

acetyl]-allosyl(1→2)glucoside*						
Isoscutellarein 7-*O*-[6'''-*O*-acetyl]-allosyl (1→2)-[6''-*O*-acetyl]-glycoside*	nd	0.0250	7.5122	12.1766	0.9417	0.0013
4'-*O*-Methylisoscutellarein 7-*O*-[6'''-*O*-acetyl]-allosyl(1→2)glucoside	nd	nd	1.0089	1.7216	0.1750	nd
**Hypolaetin derivatives**						
Hypolaetin 7-*O*-[6'''-*O*-acetyl]-allosyl(1→2)glucoside*	nd	nd	0.9276	0.5407	0.6778	nd
3'-*O*-Methylhypolaetin 7-*O*-[6'''-*O*-acetyl]-allosyl(1→2)glucoside*	nd	0.0067	4.5848	2.3223	2.3311	0.0166
4'-*O*-Methylhypolaetin 7-*O*-[6'''-*O*-acetyl]-allosyl(1→2)-[6''-*O*-acetyl]-glucoside*	nd	0.0346	1.9894	2.6334	0.4741	nd
**Chryseriol and apigenin derivatives**						
Chryseriol 7-*O*-[6'''-*O*-acetyl]-allosyl(1→2)glucoside	nd	nd	0.2624	nd	0.0817	nd
Apigenin 7-*O*-[6'''-*O*-acetyl]-allosyl(1→2)glucoside	nd	nd	0.1609	0.2171	nd	nd
Apigenin 7-(6''-*p*-coumaroylglucoside)	nd	nd	0.2210	0.3862	nd	nd
**Other**						
	nd	0.0021	nd	nd	nd	nd
	0.0854	0.0139	nd	nd	nd	nd
	nd	0.0211	nd	nd	nd	nd
	nd	0.0079	nd	nd	nd	nd
	0.0057	0.0089	nd	nd	nd	nd
	0.0190	0.0032	nd	nd	nd	nd

**the structure was confirmed with available standard, nd - not detected*

Table S2. Individual phenolic components in *in vitro* cultivated *S. scardica* preparations

S. scardica preparations<br>Extraction yield in total 33.66 %	**Hexane [mg/g DW] 1.49 %**	**Chloroform [mg/g DW] 2.56 %**	**Methanol [mg/g DW] 18.84 %**	**Ethyl acetate [mg/g DW] 1.1 %**	**Butanol [mg/g DW] 2.66 %**	**Water [mg/g DW] 7.01 %**
**Hydroxycinnamic acid derivatives**						
5-Caffeoylquinic acid*	0.0058	0.0040	0.9713	0.4406	1.1741	0.5892
Caffeic acid derivative	nd	nd	nd	0.0341	nd	nd
p-Coumaric acid*	nd	nd	nd	0.0079	nd	nd
**Phenylethanoids**						
Echinacoside	0.0142	0.0065	1.8175	nd	2.8874	0.4408
Lavandulifolioside*	0.0136	0.0083	1.3199	0.0233	2.2384	0.5057
Verbascoside*	0.0302	0.0140	4.8047	2.8714	6.8588	0.2958
Forsythoside A	0.0018	nd	nd	0.1468	0.2621	nd
Echinacoside isomer	nd	nd	nd	nd	nd	nd
Leucoseptoside A*	0.0103	nd	0.9413	nd	0.2659	0.0225
Alyssonoside	0.0208	nd	1.2153	0.2601	1.0281	0.0494
**Isoscutellarein derivatives**						
Isoscutellarein 7-*O*-allosyl(1→2)glucoside	nd	nd	0.6010	0.2318	0.2187	nd
Isoscutellarein 7-*O*-[6'''-*O*-acetyl]-allosyl(1→2)glucoside*	0.0342	0.0119	1.9341	1.6531	1.5787	nd
4'-*O*-Methylisoscutellarein 7-*O*-allosyl(1→2)glucoside	nd	nd	nd	0.1040	nd	nd
Isoscutellarein 7-*O*-allosyl(1→2)-[6''-*O*-acetyl]-glucoside	nd	nd	nd	nd	nd	nd
4'-*O*-Methylisoscutellarein 7-*O*-allosyl(1→2)-[6''-*O*-acetyl]-glucoside	nd	nd	nd	0.0796	nd	nd
4'-*O*-Methylisoscutellarein 7-*O*-[6'''-*O*-acetyl]-allosyl(1→2)glucoside*	0.0144	nd	0.9694	1.3448	0.2912	nd

Isoscutellarein 7-*O*-[6'''-*O*-acetyl]-allosyl (1→2)-[6''-*O*-acetyl]-glycoside*	nd	nd	nd	0.2038	nd	nd
4'-*O*-Methylisoscutellarein 7-*O*-[6'''-*O*-acetyl]-allosyl(1→2)glucoside	nd	nd	nd	nd	nd	nd
**Hypolaetin derivatives**						
Hypolaetin 7-*O*-[6'''-*O*-acetyl]-allosyl(1→2)glucoside*	nd	nd	0.1299	0.3115	nd	nd
3'-*O*-Methylhypolaetin 7-*O*-[6'''-*O*-acetyl]-allosyl(1→2)glucoside*	nd	nd	0.3502	0.1976	0.3786	nd
4'-*O*-Methylhypolaetin 7-*O*-[6'''-*O*-acetyl]-allosyl(1→2)-[6''-*O*-acetyl]-glucoside*	nd	nd	nd	0.0755	nd	nd
**Chryseriol and apigenin derivative**						
Chryseriol 7-*O*-[6'''-*O*-acetyl]-allosyl(1→2)glucoside	nd	nd	nd	nd	nd	nd
Apigenin 7-*O*-[6'''-*O*-acetyl]-allosyl(1→2)glucoside	nd	nd	nd	nd	nd	nd
Apigenin 7-(6''-*p*-coumaroylglucoside)	nd	nd	0.1317	0.0890	nd	nd
**Other** phenolic components						
	nd	0.4224	nd	nd	nd	nd
	nd	0.0958	nd	nd	nd	nd

**the structure was confirmed with available standard, nd - not detected*

Table S3. Regression equation, correlation coefficient (R^2), linear range, limit of detection (LOD), and limit of quantification (LOQ) for the available standards

Standard	Regression equation	R^2	Linear range (μmol/L)	LOD	LOQ
5-Caffeoylquinic acid[a]	$y = 57.19\ x - 86.36$	0.9996	3.99 – 300	1.20±0.20	4.10±0.70
Verbascoside	$y = 34.10\ x + 3.42$	0.9999	3.73 – 300	1.12±0.12	3.73±0.40
Hypolaetin 7-*O*-[6'''-*O*-acetyl]-allosyl(1→2)glucoside	$y = 55.24\ x - 77.25$	0.9988	16.98 – 300	5.10±0.44	16.98±1.47
-*O*-[6'''-*O*-acetyl]-allosyl(1→2)glucoside	$y = 38.74\ x + 62.75$	0.9997	11.02 – 300	3.31±0.32	11.02±1.07
4'-*O*-Methylhypolaetin 7-*O*-[6'''-*O*-acetyl]-allosyl(1→2)-[6''-*O*-acetyl]-glucoside	$y = 39.34\ x + 55.34$	0.9999	10.32 – 300	3.10±0.12	10.32±0.40
Isoscutellarein 7-*O*-[6'''-*O*-acetyl]-allosyl(1→2)glucoside	$y = 34.02\ x + 66.39$	0.9978	10.39 – 300	3.12±0.22	10.39±0.73
4'-*O*-Methylisoscutellarein 7-*O*-[6'''-*O*-acetyl]-allosyl(1→2)glucoside	$y = 55.32\ x + 13.79$	0.9996	3.33 – 300	1.00±0.09	3.33±0.30
Isoscutellarein 7-*O*-[6'''-*O*-acetyl]-allosyl (1→2)-[6''-*O*-acetyl]-glycoside	$y = 39.65\ x + 3.06$	0.9996	6.66 – 300	2.00±0.18	6.66±0.60
Apigenin 7-*O*-glucoside	$y = 79.34\ x - 128.41$	0.9995	17.38 – 300	5.22±0.55	17.38±1.83

[a]Means ($n = 6$)

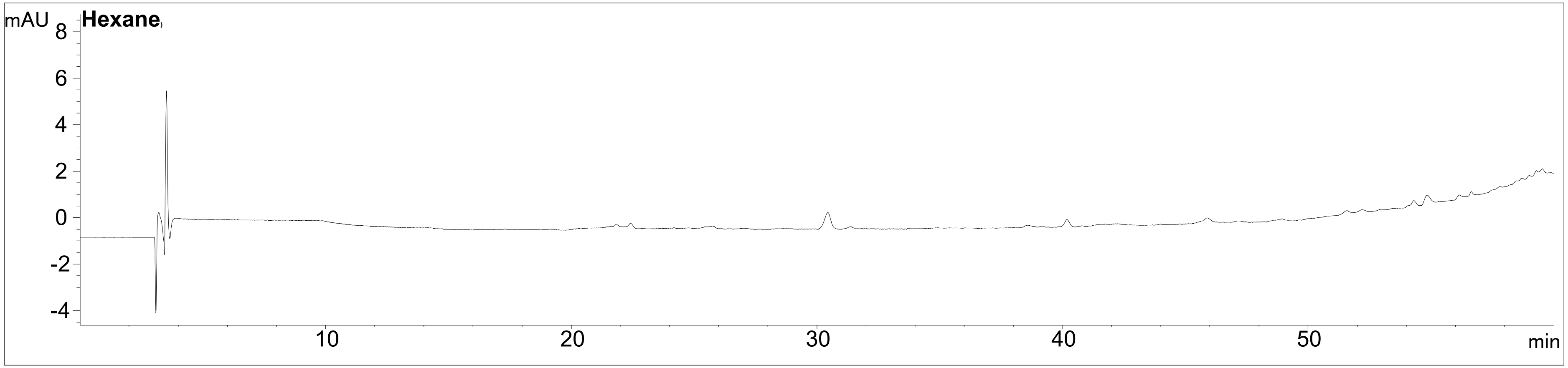
Hexane
mAU
8
6
4
2
0
-2
-4
10
20
30
40
50
min

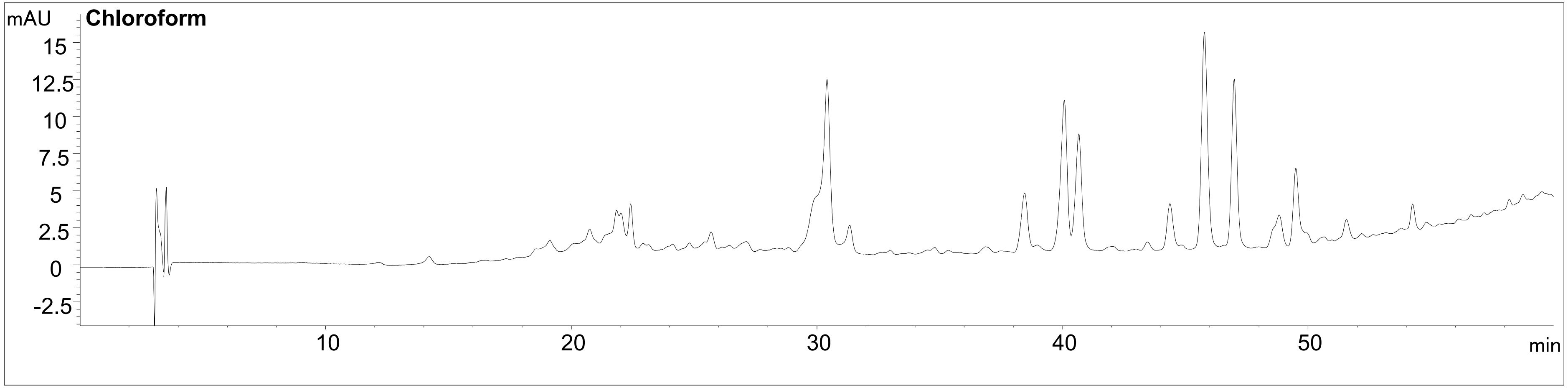
Chloroform
mAU
15
12.5
10
7.5
5
2.5
0
-2.5
10
20
30
40
50
min

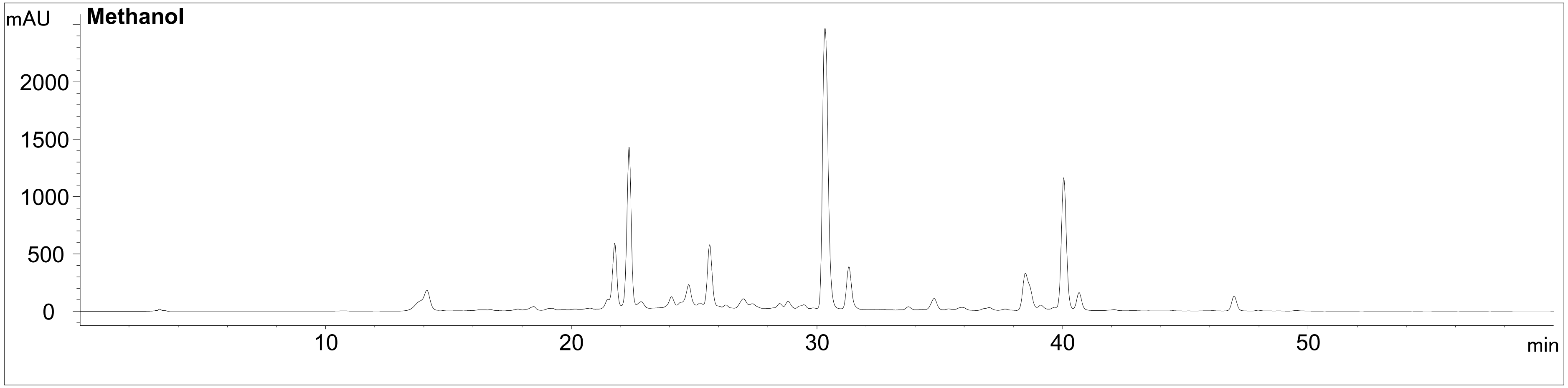
mAU
Methanol
2000
1500
1000
500
0
10
20
30
40
50
min

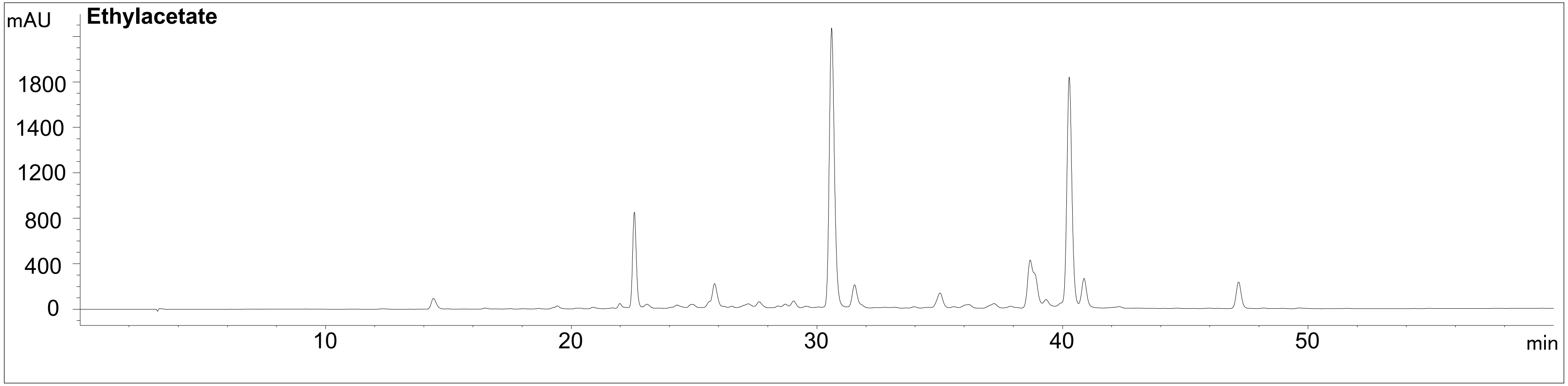
mAU
Ethylacetate
1800
1400
1200
800
400
0
10
20
30
40
50
min

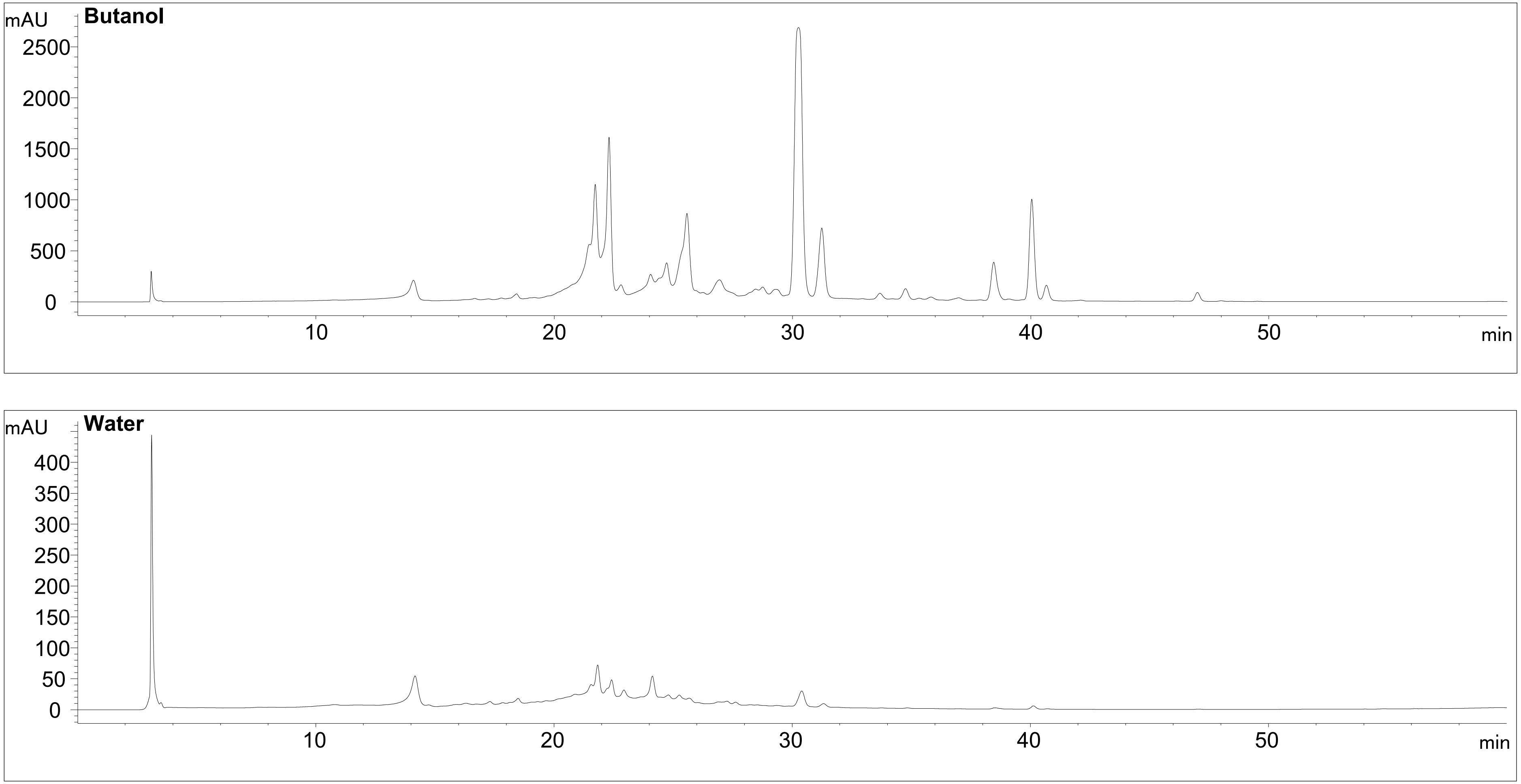

Figure S1 (a). Chromatograms of various extracts from wild growing *S. scardica* at 330 nm.

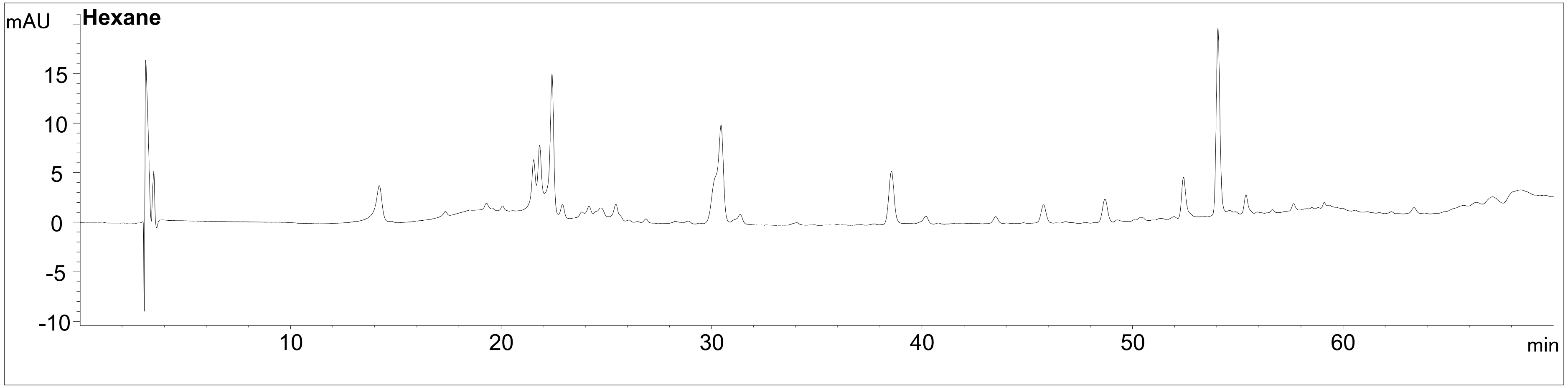
mAU
Hexane
15
10
5
0
-5
-10
10
20
30
40
50
60
min

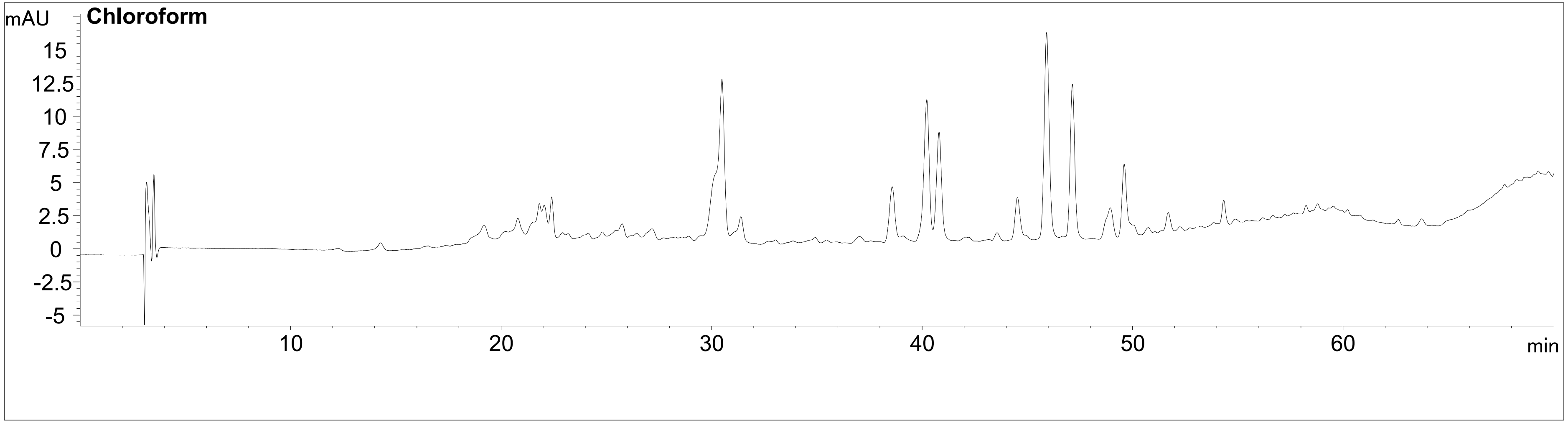
mAU
Chloroform
15
12.5
10
7.5
5
2.5
0
-2.5
-5
10
20
30
40
50
60
min

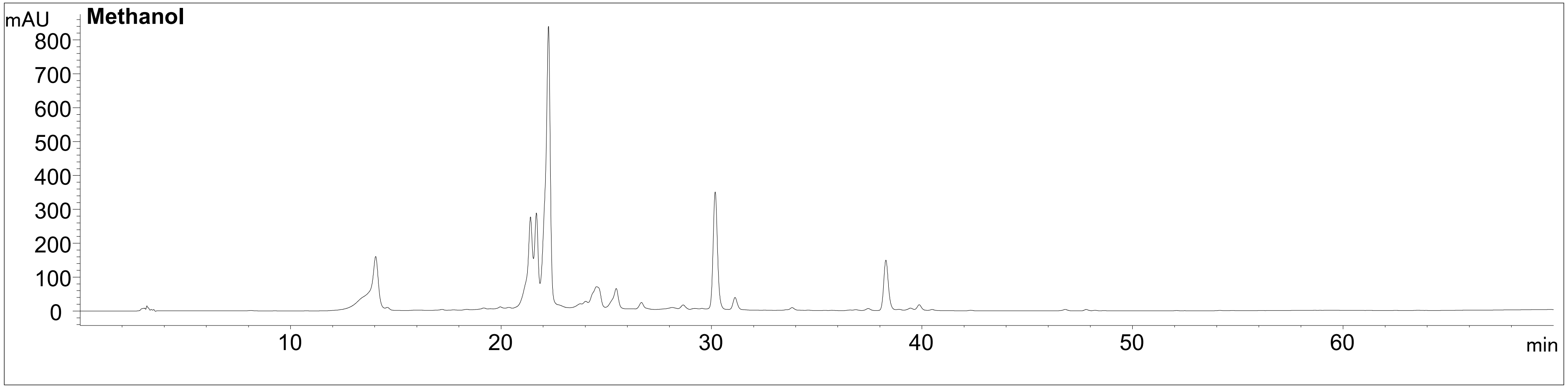
Methanol
mAU
800
700
600
500
400
300
200
100
0
10
20
30
40
50
60
min

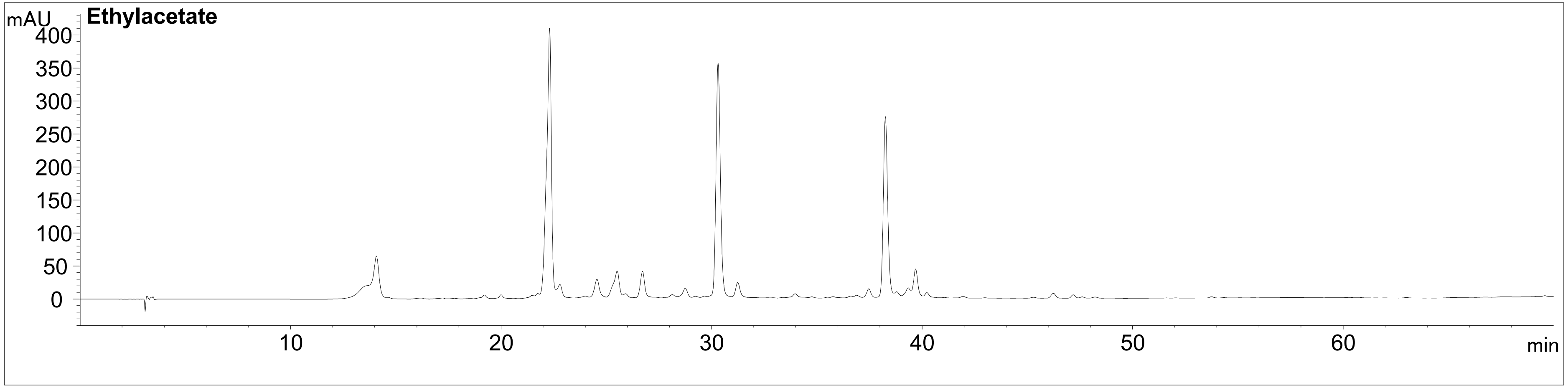
Ethylacetate
mAU
400
350
300
250
200
150
100
50
0
10
20
30
40
50
60
min

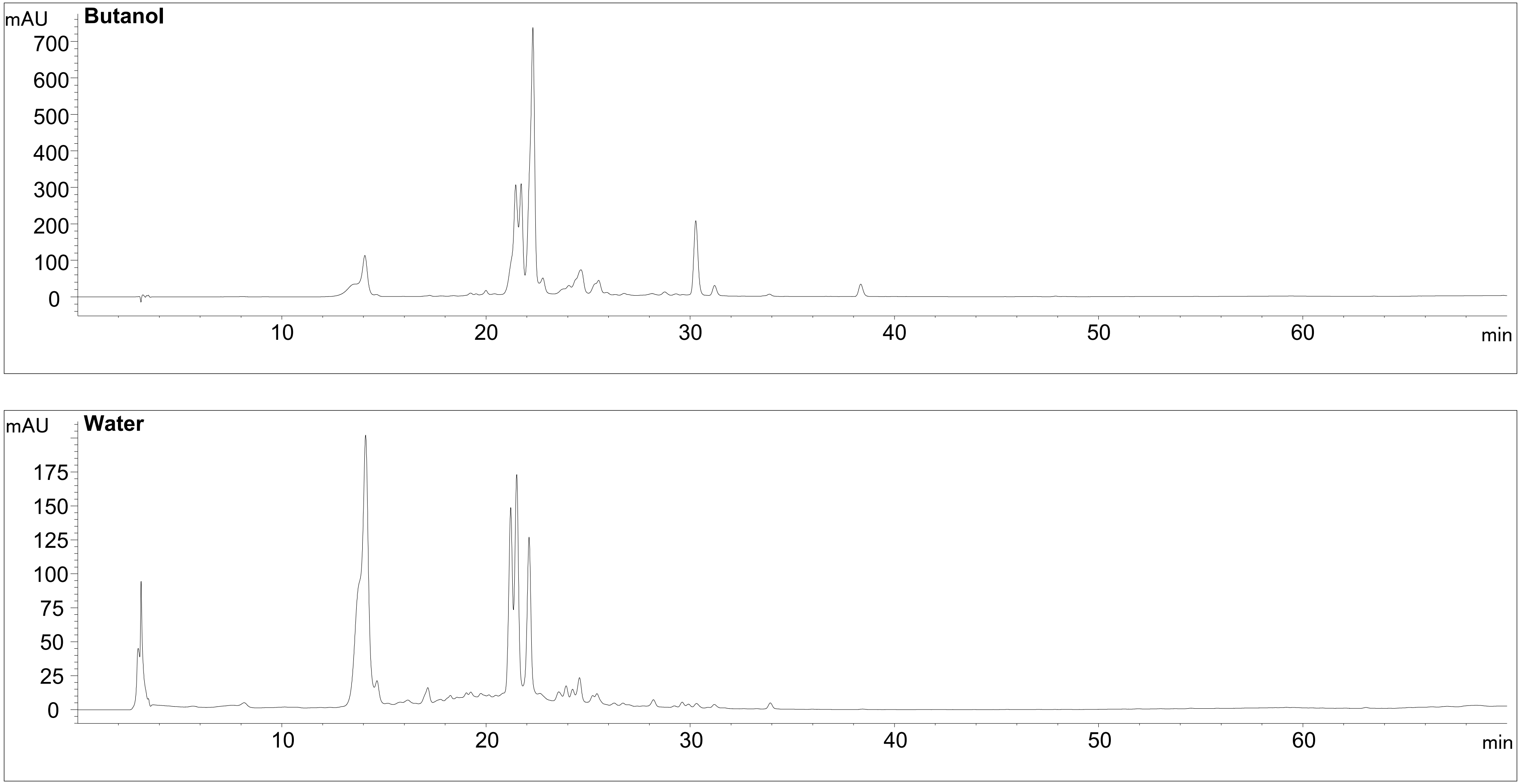

Figure S1 (b). Chromatograms of various extracts from *in vitro* cultivated *S. scardica* at 330 nm.
